# The Valorization Potential of Freshwater Fish, Including Common Carp (*Cyprinus carpio*), in the Context of Obtaining Functional Food Ingredients

**DOI:** 10.3390/foods15173027

**Published:** 2026-08-27

**Authors:** Grzegorz Tokarczyk, Katarzyna Felisiak, Grzegorz Bienkiewicz, Iwona Adamska, Sylwia Przybylska, Agnieszka Hrebień-Filisińska, Patrycja Biernacka, Hadria Boussioud

**Affiliations:** 1 Department of Fish, Plant and Gastronomy Technology, West Pomeranian University of Technology in Szczecin, Pawła VI 3, 71-459 Szczecin, Poland; 2 Department of Commodity Science, Quality Assessment, Process Engineering and Human Nutrition, West Pomeranian University of Technology in Szczecin, Pawła VI 3, 71-459 Szczecin, Poland; 3 Institute of Nutrition and Agro-Food Technologies, University of Constantine 1, Route de Aïn El Bey, P.O. Box 325, Constantine 25017, Algeria

**Keywords:** freshwater fish, functional ingredients, protein hydrolysates, extraction technologies, polyunsaturated fatty acids

## Abstract

Freshwater fish represent an underutilized source of bioactive compounds with potential applications in food enrichment and functional food design. This article reviews the current state of knowledge regarding bioactive components derived from freshwater fish, including proteins, amino acids, protein hydrolysates, bioactive peptides, lipids and polyunsaturated fatty acids, polysaccharides, pigments, minerals, vitamins, and other compounds of high functional value. Particular attention is paid to their biological and technological properties and potential applications in food products. Conventional and modern extraction and processing technologies used on an industrial scale are also discussed. Furthermore, the importance of biorefinery as a tool for the comprehensive management of freshwater fish biomass is presented. Available data indicate significant potential for bioactive components derived from freshwater fish, but their wider application requires further research to optimize recovery processes and their use in real food matrices.

## 1. Introduction

Freshwater fish play a significant role in the food economy, constituting a significant source of highly digestible animal protein with a favorable amino acid profile in the human diet. They are particularly important in regions of the world characterized by well-developed aquaculture and high availability of local raw materials, while in other areas their potential remains underutilized. Their contribution to food production is particularly significant in East, South, and Southeast Asia, where inland aquaculture is highly developed, as well as in Central and Eastern Europe, where pond-based production of common carp and other cyprinids retains considerable economic, cultural, and regional importance [1]. According to the latest statistics of the Food and Agriculture Organization of the United Nations, global aquatic animal production reached 195 million tons in 2024. Of this amount, 103 million tons were produced through aquaculture, while approximately 92 million tons originated from capture fisheries. Inland aquaculture alone supplied 64.3 million tons of aquatic animals [2]. These data demonstrate the increasing contribution of aquaculture in the global supply of aquatic foods.

In the European Union, aquaculture production amounted to approximately 1.0 million tons in 2024, representing a decrease of 3.7% compared with 2023. Poland contributed 43,554 tons, corresponding to 4.3% of total EU aquaculture production [3]. Freshwater aquaculture in Central and Eastern Europe is strongly associated with cyprinids, particularly common carp (*Cyprinus carpio*), although bream (*Abramis brama*), catfish (*Silurus glanis*), eel (*Anguilla anguilla*), grass carp (*Ctenopharyngodon idella*), pike (*Esox lucius*), pike-perch (*Sander lucioperca*), rainbow trout (*Oncorhynchus mykiss*), silver carp (*Hypophthalmichthys molitrix*) and bighead carp (*Hypophthalmichthys nobilis*) and locally also crucian carp (*Carassius carassius*), tench (*Tinca tinca*), European perch (*Perca fluviatilis*) and whitefish (*Coregonus lavaretus*) [4,5,6,7,8]. Globally, grass carp (*Ctenopharyngodon idella*), silver carp (*Hypophthalmichthys molitrix*), bighead carp (*Hypophthalmichthys nobilis*), common carp (*Cyprinus carpio*), Indian major carps, tilapia, pangasiid catfish, and salmonids are among the major freshwater fish groups produced for human consumption [2].

Freshwater fish should be considered not only as a dietary protein source, but also as a raw material rich in compounds with potential biological and technological functionality. Edible tissues and processing side streams, including skin, scales, heads, frames, bones, viscera, and roe, may contain bioactive peptides, collagen, gelatin, enzymes, unsaturated fatty acids, pigments, vitamins, minerals, and glycosaminoglycans. Fish-derived constituents have been associated with antioxidant, anti-inflammatory, antihypertensive, and immunomodulatory properties, as well as those supporting the functioning of the cardiovascular and osteoarticular systems. However, the strength of evidence varies considerably among compounds and frequently originates from in vitro or preclinical studies [9,10]. Their recovery may additionally reduce nutrient losses and support the development of value-added ingredients within circular and biorefinery-based processing systems [10,11].

Despite this potential, freshwater fish have received less integrated attention than marine seafood in reviews concerning bioactive ingredients and food applications. Existing publications frequently combine marine and freshwater species or focus on a single compound class, processing sidestream, or extraction technique. Consequently, knowledge regarding the relationships among freshwater species, tissue sources, composition, recovery methods, processing stability, sensory effects, safety, and performance in food matrices remains fragmented. This gap is particularly evident for common carp, for which the nutritional composition and production importance are relatively well documented, whereas direct applications of carp-derived bioactive fractions in fortified or functional foods remain limited.

The aim of this review is therefore to evaluate carp as a primary freshwater fish species, in the context of other freshwater fish species for which sufficient data are available. This review discusses the main groups and sources of bioactive compounds, their biological and technological properties (in the context of food processing), and conventional, enzymatic, fermentation-based, and modern extraction technologies suitable for industrial-scale application. Particular attention is paid to ingredient stability, sensory compatibility, safety, regulatory issues, integration with biorefineries, and the current lack of research on application in real food systems. This approach aims to identify the most promising opportunities and key research needs related to the responsible use of freshwater fish biomass for the production of high-value food ingredients.

## 2. Sustainable Development and Circular Economy

Sustainable development is a form of managing natural resources, taking into account their natural depletion. Aquaculture cannot be regarded as an inherently or universally sustainable method of food production. Its environmental performance depends on the cultured species, farming intensity, feed composition and conversion efficiency, energy source, water and land requirements, stocking density, and treatment of nutrient-rich effluents [12,13,14,15]. Potential environmental burdens include eutrophication caused by uneaten feed and fish excreta, greenhouse gas emissions, consumption of water and energy, dependence on externally produced feed ingredients, chemical and veterinary inputs, disease transmission, and the escape of cultured fish into natural ecosystems [16]. This is perfectly illustrated by the Green and Blue Economy model, which involves the sustainable management of land resources (Green Economy) and water resources (Blue Economy), ultimately stimulating economic growth while maintaining the health of all ecosystems.

Carp production illustrates the need for sustainability assessments that take into account the specificities of the given context. Life-cycle analyses indicate that traditional pond-based carp production may have a lower climate change impact than energy-intensive recirculating systems and several terrestrial animal production systems. Nevertheless, pond aquaculture may still contribute to nutrient emissions, water and land occupation, and other environmental impacts. Moreover, organic production does not necessarily perform better than conventional production in every impact category [14]. The sustainability of carp aquaculture should therefore be evaluated using multiple environmental, economic, and social indicators rather than assumed based on the production system alone [16,17,18,19].

Circular economy strategies can improve resource efficiency in freshwater aquaculture by extending biomass utilization beyond the edible part of the carcass (the fillet). Processing of freshwater fish such as carp, trout, pike, and bream generally generates approximately 40–60% of the original biomass as heads, frames, bones, skin, scales, fins, viscera, blood, and trimming residues [11]. The proportion depends on the species, body size, anatomical characteristics, processing method, and final product. In common carp, trimmed fillet yields of approximately 34–39% have been reported, indicating that the non-fillet fraction can exceed 60% [20]. However, this fraction should not be treated uniformly as waste because many of its components remain suitable for direct food use or further processing.

From a food-enrichment perspective, the highest priority should be given to preserving edible material and recovering food-grade ingredients before directing the remaining fractions toward feed, fertilizer, bioenergy, or other lower-value applications. Common carp skin, scales, bones, frames, heads, and viscera may provide proteins, protein hydrolysates, bioactive peptides, collagen, gelatin, lipids, enzymes, minerals, and glycosaminoglycans. Their recovery can increase the value obtained from each fish while reducing the loss of nutritionally and technologically useful constituents [13,21,22]. A cascading biorefinery approach may therefore involve the initial recovery of oxidation-sensitive lipids and other high-value compounds, followed by the isolation or enzymatic conversion of proteins and connective-tissue components, mineral recovery, and, finally, biological or energetic treatment of residues that cannot be used in food [13,18,19].

Valorization should not automatically be equated with environmental sustainability. Extraction and purification may require substantial quantities of water, energy, enzymes, solvents, and auxiliary materials, while drying and stabilization can further increase production costs and environmental burdens. The feasibility of recovering bioactive ingredients consequently depends on the scale and continuity of the raw material supply, compositional variability, process yield, product purity, market value, and available processing infrastructure [12,19,21]. Life-cycle assessment and techno-economic analysis should therefore accompany process development to identify possible burden shifting and determine whether the recovered food ingredient provides a genuine environmental and economic advantage.

Accordingly, a circular model for freshwater fish should integrate responsible aquaculture production, maximization of edible yield, source separation of processing by-products, recovery of food-grade bioactive ingredients, and appropriate use of the residual biomass. In the case of carp, this approach offers an opportunity to expand the currently limited range of processed products and to develop ingredients for food fortification. However, its successful implementation requires more species-specific data on biomass availability, extraction yields, ingredient functionality, consumer acceptance, regulatory status, and environmental performance in industrial-scale systems.

## 3. Bioactive Compounds Important for Food Enrichment

### 3.1. Protein

#### 3.1.1. Nutritional Proteins and Amino Acids

Fish are a rich source of protein. Protein content depends on the species, state, season, and part of the fish (Table 1). From a nutritional perspective, the amount of muscle tissue determines its utility. Freshwater fish, unlike saltwater fish, are usually sold as carcasses, less often as fillets, due to the high proportion of viscera and bones. However, ready-made meals, which require pre-processing, are becoming increasingly popular. Therefore, fillets, both skin-on and skinless, as well as mechanically deboned meat, are used. Despite the high yields of the latter method, the amount of by-products ranges from 30% to 70% [23]. Nitrogen compounds that can be recovered from fish by-products are, among others, proteins, protein hydrolysates, peptides and amino acids, and active enzymes and collagen.

Fish are rich in valuable proteins of good digestibility and accessibility. The total protein content of freshwater fish ranges from about 15 to 21% [24], but varies between species. Carp (*Cyprinus carpio*) meat typically contains about 15–19% protein [25,26]. The food value of freshwater fish proteins is determined not only by their total concentration but also by their amino acid composition, digestibility, and technological behaviour [27]. Common carp muscle contains myofibrillar proteins, predominantly myosin and actin, as well as sarcoplasmic and stromal proteins [28]. Myofibrillar proteins are particularly important for gel formation, emulsification, water retention, and texture development in processed foods. Sarcoplasmic proteins contribute to nutritional quality but may also affect colour, oxidation, and flavour, whereas stromal proteins, principally collagen, determine the properties of connective tissues [29,30]. Fish proteins are easily digestible, high-value, containing all essential amino acids, with a very high lysine content. In freshwater fish, high levels of lysine (Lys) and glutamic acid (GLU), e.g., 5–6 g/100 g Lys and 7.5–9 g/100 g Glu, respectively [25,31,32]. Freshwater fish are also a rich source of collagen. Skin, bones, and scales constitute as much as 15% of the byproducts, so attempts to obtain collagen and peptide hydrolysates have been conducted for years, with the use of scales and bones [32,33].

**Table 1:** Representative protein-based compounds recovered from common carp and other freshwater fish, their production methods, principal findings, and limitations.

Species and Material	Protein-Based Fraction	Production or Recovery Method	Principal Finding	Food Relevance and Principal Limitation	Ref.
Common carp (*Cyprinus carpio*) muscle	Muscle proteins and amino acids	Conventional compositional analysis	Muscle generally contains approximately 15–19% protein and all indispensable amino acids	Nutritionally valuable protein source; composition varies with farming and biological factors	[25]
Common carp muscle	Acidic and alkaline protein isolates	pH-shift solubilization followed by precipitation near pH 5.5	Protein recovery reached approximately 76.3% for the acidic process and 87.6% for the alkaline process	High recovery and favourable digestibility; extreme pH may alter gelation, solubility, and oxidation stability	[34]
Tra catfish (*Pangasius hypophthalmus*) processing side streams	Fish protein isolates	pH-shift processing	Protein recovery of 83.0–88.9%, with substantial removal of lipid and ash	Demonstrates applicability to heterogeneous side streams; functionality depends on source material and process severity	[35]
Common carp bones	Acid-soluble collagen	Acid extraction after pretreatment	Collagen yield of approximately 5.09% on a dry-weight basis	Potential food-grade collagen source; relatively low thermal stability must be considered	[36]
Common carp skin, scales, and bones	Type I collagen	Acid extraction and structural characterization	Tissue-dependent yield and a denaturation temperature of approximately 28 °C	Suitable for low-temperature formulations; limited stability in heat-processed foods	[37]
Common carp scales	Hydrolysed collagen	Combined enzymatic hydrolysis	Demonstrated feasibility of producing soluble collagen hydrolysate from scales	Supports side-stream valorization; food-matrix performance and sensory effects require evaluation	[32]
Common carp processing by-products	Collagen-derived peptide fractions	Enzymatic hydrolysis and peptide fractionation	Fractions displayed antioxidant and selected techno-functional properties	Potential antioxidant or functional ingredient; evidence predominantly in vitro	[38,39]
Common carp skin gelatin	Gelatin hydrolysates and antioxidant peptides	Enzymatic hydrolysis under different process conditions	Antioxidant activity depended strongly on hydrolysis conditions; active peptides were subsequently identified	Process can be tailored to obtain functional peptides; bitterness and gastrointestinal stability remain concerns	[38,39]
Common carp skin gelatin	Protein hydrolysate	Enzymatic hydrolysis followed by administration to rats	Changes in selected oxidative stress and blood biomarkers were observed	Provides animal-level evidence, but does not establish efficacy in humans or fortified foods	[40]
Silver carp (*Hypophthalmichthys molitrix*) skin and other side streams	Gelatin	Acid/thermal conversion of collagen	Tissue source significantly influenced structure, rheology, and emulsifying properties	Demonstrates that gelatins from different tissues cannot be treated as equivalent ingredients	[31]
Silver carp surimi-processing side streams	Protein hydrolysates	Enzymatic hydrolysis followed by incorporation into surimi	Hydrolysates reduced protein oxidation and improved selected gel properties	Direct evidence of functionality in a food matrix; sensory and storage optimization still required	[41]
Crucian carp (*Carassius carassius*) muscle	Lipase-inhibitory protein hydrolysate	Alkaline protease, pH 11, 39 °C, 122 U/mL, 10 h	Noncompetitive pancreatic lipase inhibition; (K_i) = 84.13 mg/mL	High concentrations and incomplete dose reporting limit conclusions regarding practical efficacy	[42]
Bighead carp (*Hypophthalmichthys nobilis*) muscle	Bioactive peptide fractions	Enzymatic hydrolysis, fractionation, and peptide identification	Antioxidant, ACE-inhibitory, and DPP-IV-inhibitory activities were reported	Multifunctional in vitro activity; bioavailability and food application remain unconfirmed	[43]
Silver carp swim bladder	DPP-IV-inhibitory peptides	Enzymatic hydrolysis and peptide identification	Peptides with DPP-IV-inhibitory activity were identified	Potential metabolic health application; evidence remains preclinical	[44]
Sturgeon skin	ACE-inhibitory peptides	Enzymatic hydrolysis and peptide identification	Peptides with ACE-inhibitory activity were obtained	Promising antihypertensive potential; efficacy in food and humans requires validation	[45]
Yellow catfish (*Pelteobagrus fulvidraco*) skin mucus	Pelteobagrin antimicrobial peptide	Isolation and purification from mucus	Activity against Gram-positive and Gram-negative bacteria and fungi	Potential antimicrobial template rather than an established food ingredient	[46]
Tilapia (*Oreochromis mossambicus*) tissues	Hepcidin peptides	Isolation, expression analysis, and antimicrobial testing	Three hepcidins with antimicrobial functions were characterized	Relevant to discovery of antimicrobial peptides; production, safety, and food regulatory status remain unresolved	[47]

ACE—angiotensin-converting enzyme; DPP-IV—dipeptidyl peptidase-IV; (K_i)—enzyme-inhibition constant.

#### 3.1.2. Recovery and Functionality of Freshwater Fish Protein Isolates

During processing, high-quality fish parts are obtained, as well as high-protein portions that are more difficult to recover. During preliminary processing, what remains are primarily backbones and bones with remnants of meat, heads, fins, skin, scales, and viscera. All of these by-products contain significant amounts of either high-quality proteins, including collagen.

The method of protein recovery varies depending on the properties of the proteins. The simplest method of recovering meat tissue from freshwater fish carcasses is separation using a screw separator and a strainer. The resulting mechanically deboned meat contains all native proteins, including myofibrillar proteins. However, proteins are still present in the by-products of this processing, so collagen, other proteins, or protein hydrolysates could be obtained [34,41]. Isoelectric solubilization/precipitation, also described as pH-shift processing, which enables the recovery of muscle proteins from complex fish matrices. The material is first homogenized with water, after which the pH is adjusted to strongly acidic or alkaline conditions. At pH values distant from the proteins’ isoelectric region, the net molecular charge increases, promoting electrostatic repulsion, tissue disintegration, and solubilization of myofibrillar and sarcoplasmic proteins [48]. Insoluble components, including bones, scales, connective-tissue fragments, and some membrane material, are subsequently removed by centrifugation. Depending on matrix composition and process design, part of the lipid fraction may also be separated during this stage. The pH of the protein-containing supernatant is then adjusted to approximately 5.2–5.5, close to the isoelectric point of many fish muscle proteins. The reduction in net charge decreases protein solubility and causes precipitation. The precipitated fraction is recovered by centrifugation and may subsequently be washed and neutralized before drying or incorporation into food [34,35]. The obtained fish protein isolates contain more than 90% protein in dry matter, and the properties differ depending on the applied method (solubilization at pH 2.5 or 12.5). In common carp muscle, the alkaline and acidic variants of pH-shift processing resulted in protein recovery yields of approximately 87.6% and 76.3%, respectively. Protein isolates obtained through both routes retained a favourable amino acid profile and displayed high in vitro digestibility [34].

#### 3.1.3. Collagen and Gelatin

Collagen is usually obtained from deproteinated bones or skins by solubilization in acid; after precipitation and dialysis, it can be lyophilized [49]. In the case of common carp (*Cyprinus carpio*), the yield of collagen obtained from backbones was 5.09% based on dry matter. In the case of fish skin, the yield of collagen is higher, 12.1–13.8% for snakehead fish (*Channa striata*) skin [50], tra catfish (*Pangasius hypophthalmus*) [35], and tilapia (*Oreochromis mossambicus*) [47], or even 15.5–16.0% in the case of black carp (*Mylopharyngodon piceus*) skin and clown knifefish (*Chitala ornata*) skin [51,52]. However, because of the amount, backbones are an important source of collagen. The quality of fish collagen depends on many factors, including fish species, age, and climate, etc. The amount of amino acids (proline and hydroxyproline) influences the thermal stability of collagen, with a higher denaturation temperature for higher levels of Pro and Hyp [48]. Dikel and Yanar (2025) found the ratio of imino acids in collagen from common carp as 27.65%, and it was higher than in collagen from tilapia (*Oreochromis mossambicus*) (25.4%) [49]. Duan et al. (2009) suggested that the temperature of collagen denaturation was correlated with the ratio of amino acids, and they found that the denaturation temperature of collagen from carp skin, bones, and scales was 28 °C, while from cod skin, it was only 15 °C, so the properties of freshwater fish collagen seem to have better gelling properties [36].

Collagen is usually obtained either as gelatin or as collagen hydrolysates after enzymatic proteolysis [36,37,53]. Proteins are primarily a source of bioactive compounds, released during digestion or in vitro hydrolysis [37,39,54]. The resulting peptides are characterized by high biological value, particularly antioxidant and antiatherosclerotic, antihypertensive (ACE inhibition), antimicrobial, anti-inflammatory, anticoagulant, immunomodulatory, and antithrombotic properties [55,56,57,58].

#### 3.1.4. Protein Hydrolysates and Bioactive Peptides

There are many studies on the possibility of obtaining fish hydrolysates from by-products, most of which are focused on marine species, because processing of marine fish generates most of the by-products [59]. However, freshwater fish are becoming more and more important because of increasing aquaculture [60,61,62]. Fish protein hydrolysates are complex mixtures of peptides and free amino acids generated through chemical, microbial, or enzymatic hydrolysis [57]. Fish protein hydrolysates (FPH) could be produced by a chemical method (with concentrated acid, which causes degradation of tryptophan and lowers the amount of some other amino acids), but modern methods are focused on enzymatic proteolysis. The composition and properties of fish protein hydrolysates differ depending on species, by-products, and enzymes used. Usually, proteolytic enzymes of microbial origin (Alcalase Novozymes Corp., Merck KGaA, Darmstadt, Germany, Neutrase Novozymes A/S, Bagsvaerd, Denmark, Protamex Novozymes Corp., Merck KGaA, Darmstadt, Germany, alkaline proteinase), as well as plant origin (Papain, Bromelain), or animal origin (Pepsin, Trypsin), and mixtures of enzymatic preparations [26,36,41,63,64].

Fish protein hydrolysates usually show antioxidant properties because of the content of peptides containing hydrophobic amino acids, lysine, and cysteine, etc. [32,38,39,40,56,65]. Prevention of oxidative stress is one of the reasons for anti-cancer activity. FPHs obtained with Alcalase from Asian swamp eel (*Monopterus* sp.) flesh demonstrated anti-proliferative activity against the human breast cancer cell line (MCF-7 Merck KGaA, Darmstadt, Germany) [62]. Sturgeon (*Acipenser schrencki*) skin hydrolysate contains peptides of ACE inhibitory activity, as well as bighead carp meat hydrolysate [45,66].

The biological and technological properties of a hydrolysate depend on the substrate, enzyme specificity, enzyme-to-substrate ratio, pH, temperature, hydrolysis time, and degree of hydrolysis [62]. Limited hydrolysis can improve solubility, emulsification, and digestibility, whereas extensive hydrolysis usually produces smaller peptides with increased antioxidant activity but may reduce emulsifying capacity and generate bitterness through the exposure of hydrophobic amino acid residues [62,67]. Consequently, the hydrolysis conditions must be optimized for a defined application rather than solely for maximal peptide yield.

Liu et al. optimized alkaline protease hydrolysis of water-soluble protein from crucian carp (*Carassius carassius*) muscle at pH 11, 39 °C, an enzyme dosage of 122 U/mL, and a hydrolysis time of 10 h. The resulting hydrolysate inhibited porcine pancreatic lipase through a noncompetitive mechanism, with a reported (Ki) of 84.13 mg/mL. Although the authors also reported a maximal inhibition value of 53.04%, this endpoint was not unambiguously linked to a defined hydrolysate concentration. The relatively high concentrations used in the kinetic experiments and the absence of validation in a food or in vivo model limit conclusions regarding practical effectiveness in combating obesity [42]. In turn, hydrolysates of tilapia meat showed DPP-IV (Merck KGaA, Darmstadt, Germany) inhibitory activity and caused an increase in insulin secretion and glucose tolerance [66]. A similar effect was found in the case of hydrolysates from tilapia (*Oreochromis* sp.) scales [67] and bighead carp (*Hypophthalmichthys nobilis*) muscles [43]. These effects could be caused by peptides containing 3–15 amino acids, especially with proline, leucine, isoleucine, valine, glycine, and phenylalanine residues [44]. However, animal evidence does not directly establish efficacy in humans or demonstrate that comparable effects will be retained after incorporation into food.

Moreover, fish hydrolysates might show anti-microbial effects. Peptides of anti-microbial activity usually contain between 10 and 50 amino acids with a net positive charge of 2 to 8. Peptides obtained from freshwater fish intestines, skins, heads, muscles, etc., or from skin mucosa can have anti-microbial effects [46,47].

### 3.2. Lipids and Lipid-Derived Bioactive Compounds

#### 3.2.1. Lipid Classes

Fatty acids in fish tissues occur predominantly in esterified form as constituents of neutral and polar lipids. Neutral lipids comprise mainly triacylglycerols, together with smaller quantities of diacylglycerols, monoacylglycerols, sterol esters, free sterols, and hydrocarbons. Triacylglycerols represent the principal energy-storage fraction and usually increase with total tissue fat content. Polar lipids are structural components of cellular membranes and include phosphatidylcholine, phosphatidylethanolamine, phosphatidylserine, phosphatidylinositol, sphingolipids, lysophospholipids, and glycolipids. Cholesterol and other unsaponifiable compounds, including squalene, constitute additional lipid-derived components of potential nutritional or technological interest [68,69].

One of the greatest benefits of fish meat is its content of valuable lipids, rich in n-3 polyunsaturated fatty acids (PUFAs), with particular emphasis on eicosapentaenoic acid (EPA, 20:5 n-3) and docosahexaenoic acid (DHA, 22:6 n-3) [70]. Polyunsaturated fatty acids (PUFAs) are generally classified into two main categories: the α-linolenic acid (ALA) family, or n-3 (omega-3) fatty acids, in which the first unsaturated bond is located at the third carbon atom; the linoleic acid (LA) family, or n-6 (omega-6) fatty acids, characterized by the presence of the first double bond at the sixth carbon atom [71].

Both ALA and LA are classified as essential fatty acids (EFAs), which results from the human body’s inability to synthesize them endogenously [72]. After being supplied with food, both compounds undergo further metabolic pathways, serving as substrates for the production of further polyunsaturated fatty acids. As a result of the elongation and desaturation of ALA, eicosapentaenoic acid (EPA; 20:5, n-3) and docosahexaenoic acid (DHA; 22:6, n-3) are synthesized. Analogous LA transformations lead to the formation of derivatives from the n-6 group, including γ-linolenic acid (GLA) and arachidonic acid [73]. It is worth emphasizing that the metabolism of both families is based on the same enzymatic apparatus, which generates competition between ALA and LA for the same enzymes, affecting the final lipid profile [74]. Increasing the amount of omega-6 in the diet shifts the balance in favor of arachidonic acid (AA), reducing the level of long-chain omega-3 acids [75,76] milies is based on the same enzymatic apparatus, which generates competition between ALA and LA for the same enzymes, affecting the final lipid profile [74]. Increasing the amount of omega-6 in the diet shifts the balance in favor of arachidonic acid (AA), reducing the level of long-chain omega-3 acids [77]. The efficiency of biosynthesis of long-chain EPA and DHA from α-linolenic acid is severely limited in humans—conversion to EPA is only 8–12%, while conversion to DHA drops below 1% [73]. For this reason, it is crucial to directly provide the entire spectrum of these acids, including ALA, EPA, and DHA, in the daily diet [78]. Although observational studies frequently associate fish consumption with lower cardiovascular risk, evidence from randomized trials of n-3 fatty acid supplementation is inconsistent. In the VITAL trial, marine n-3 fatty acid supplementation did not significantly reduce the primary composite cardiovascular endpoint in a general primary-prevention population, while the STRENGTH trial found no reduction in major adverse cardiovascular events with a high-dose EPA/DHA formulation in statin-treated high-risk patients. Similarly, the OMEMI trial reported no significant cardiovascular benefit of EPA/DHA supplementation in elderly patients after myocardial infarction [78,79,80]. These findings indicate that n-3 supplementation should not be regarded as cardioprotective With the unclear benefit of OM3FA supplementation and possibility of harm, the current data suggest that the risk of routine use of OM3FA outweighs the possibility of reduction in risk of cardiovascular event [81]. In contrast, REDUCE-IT demonstrated a reduction in cardiovascular events with icosapent ethyl, a highly purified EPA preparation, indicating that clinical outcomes may depend on the chemical form, dose, formulation, and characteristics of the target population [82].

The parent lipid class and the stereospecific position of a fatty acid on the glycerol backbone may influence digestion, absorption, oxidative stability, membrane incorporation, and technological behavior. In phospholipids, long-chain PUFA may occur at the sn-1 or sn-2 position, although unsaturated fatty acids are frequently enriched at sn-2. However, species- and tissue-specific information concerning the positional distribution of EPA and DHA in freshwater fish remains limited.

The distribution of neutral and polar lipids is strongly tissue-dependent. In common carp, muscle lipids were reported to consist predominantly of triacylglycerols, whereas phospholipids represented the major lipid fraction in male and female gonads [74]. In a comparison of several freshwater species, neutral lipids accounted for 71.5–93.3% of total lipid extracts from meat, heads, and viscera, while common carp muscle contained a substantial glycolipid fraction [75]. These results indicate that total lipid content cannot serve as a direct indicator of the amount of polar lipids. Lean muscle generally contains a greater relative proportion of membrane-associated polar lipids, whereas fatty tissues contain more storage triacylglycerols; nevertheless, the absolute quantity of each fraction also depends on species, tissue, physiological status, and feeding regime [83].

#### 3.2.2. Diversity of Lipid Profiles Among Freshwater Fish

Freshwater fish differ significantly in terms of total lipid content and composition. Variation occurs not only between species but also within the same species according to diet, trophic position, farming system, season, sex, age, body size, reproductive status, water temperature, and anatomical location [84,85,86,87,88,89,90]. Fixed classification of a species as lean, medium-fat, or fatty may therefore be misleading when values are compiled from different production systems and sampling periods.

Due to their proven health-promoting effects, n-3 fatty acids are recommended by international and national institutions dealing with nutrition, public health, and scientific societies. Eating fish at least twice a week and supplementing with fish oils are recommended, wherein oils are extracted from fish flesh or the liver of some species and are the basis for the production of more concentrated pharmaceutical-grade EPA and DHA preparations [91]. Oils can also be a source of n-3 fatty acids in the production of functional foods. These are typically obtained by fortifying traditional foods with fish oils, using both unmodified and modified oils, such as emulsified, microencapsulated [92,93], and oleogels [94].

Lipids in fish meat determine not only its health-promoting properties but also its tastiness, technological suitability, and susceptibility to oxidation [95]. Fat is considered the third major component of fish muscle (along with water, moisture, and protein), located primarily in the subcutaneous tissue, skin, liver, muscle tissue, mesenteric tissue, belly flap, and head [96]. Directly beneath the skin along the side of the body (and in some active species also in the waist near the spine), red muscles are found, which are typically richer in lipids (and nucleic acids, myoglobin, and vitamin B) than white muscles [97]. However, it is believed that fish skin typically contains higher amounts of lipids than muscle tissue. There is a clear relationship between moisture and fat; fish with lower moisture content tend to have higher fat and protein content. Furthermore, fat content usually decreases from head to tail. The fat content of fish can typically range from 0.2% to about 20% [96]. For example, in rainbow trout, reported values in the present dataset range from 2.06% to 11.41% total fat (Table 2). Farmed rainbow trout generally belongs to the medium-fat or fatty category, although leaner values may occur depending on the sampled tissue and production conditions [21,92]. Similar variability is evident in common carp, for which reported fat concentrations range from less than 1% to more than 6%. These broad ranges justify presenting species data as intervals accompanied by information on tissue, season, diet, and production system.

Common carp is particularly responsive to dietary lipid supply. Its muscle commonly contains oleic, palmitic, linoleic, α-linolenic, arachidonic, EPA, and DHA, but their relative proportions vary greatly. Pond-reared carp receiving cereal-based supplementary feed may accumulate considerable quantities of oleic and linoleic acids, while access to zooplankton and other natural pond organisms can increase the supply of EPA and DHA. Common carp can elongate and desaturate C18 precursors, but the contribution of this pathway to tissue EPA and DHA depends on precursor availability, competition between n-3 and n-6 substrates, and physiological demand [90,105,109]. Therefore, the lipid quality of carp should be evaluated on the basis of actual production conditions rather than species identity alone.

Compared with many marine species, freshwater fish generally contain more linoleic acid, α-linolenic acid, and arachidonic acid and often have a lower proportion of EPA and DHA [82]. Cold-water or predatory freshwater species, including pike, pikeperch, perch, and some salmonids, may contain substantial EPA+DHA concentrations. Conversely, farmed fish supplied with feeds rich in terrestrial plant oils may contain lower proportions of long-chain n-3 PUFA than wild conspecifics [85,86,88,101,110]. A comparison based solely on average values for freshwater and marine environments therefore masks significant variation depending on the species and production method.

Freshwater fish also differ in the allocation of lipids among anatomical compartments. Muscle, skin, liver, visceral adipose tissue, gonads, heads, and viscera may have distinct lipid-class and fatty acid profiles. In common carp, gonads provide a comparatively polar-lipid-rich matrix, while viscera and abdominal deposits contain larger quantities of neutral storage lipids [74]. Livers of several freshwater species can be important sources of EPA and DHA, but their use for food ingredients requires stringent contaminant and vitamin-safety assessment. Processing side streams should therefore be separated according to tissue origin rather than combined into a heterogeneous lipid source [111].

#### 3.2.3. Polar Lipids and Other Bioactive Lipid Classes

Polar lipids deserve greater attention because their functionality is not determined solely by the fatty acids they contain. Phosphatidylcholine and phosphatidylethanolamine contribute to membrane structure and may improve the dispersion of hydrophobic compounds in aqueous food systems. Phosphatidylserine, phosphatidylinositol, lysophospholipids, and sphingolipids may also exhibit specific biological activities, although their occurrence and molecular composition in common carp have not been characterized as extensively as total fatty acids [112,113].

The amphiphilic structure of phospholipids provides technological functions relevant to food enrichment, including emulsification, interfacial stabilization, and the formation of liposomes or other delivery systems [114]. However, phospholipids enriched in long-chain PUFA may be highly susceptible to oxidation because the unsaturated acyl chains are located near membrane interfaces where pro-oxidative metals, oxygen, and water-soluble initiators may be present [25]. Assessment of a freshwater fish polar-lipid fraction should therefore include molecular composition, oxidation stability, emulsifying properties, residual protein content, and sensory quality.

Glycolipids are another insufficiently examined fraction. A comparatively high proportion of glycolipids has been reported in common carp muscle lipid extracts [75]. Nevertheless, the specific glycosphingolipid and glyceroglycolipid species responsible for this fraction, their bioavailability, and their functionality in food matrices remain largely unknown [115]. Modern lipidomic methods could help distinguish genuinely bioactive molecular species from broad gravimetric lipid classes.

Freshwater fish may also provide minor fatty acids. Furan fatty acids have been identified in common carp muscle and gonadal lipids [74]. These compounds have attracted interest because of their possible radical-scavenging and anti-inflammatory properties, but their concentrations are low, their biological significance remains incompletely established, and oxidation or processing may modify their abundance.

#### 3.2.4. Relevance to Food Enrichment and Processing Stability

The selection of freshwater fish lipids for food enrichment should consider the complete lipid matrix rather than EPA+DHA content alone. Neutral-lipid-rich oils may be appropriate for bulk enrichment, whereas phospholipid-rich fractions may offer emulsifying functionality and alternative delivery forms. The tissue source is therefore important: visceral and abdominal fat may provide higher extraction yields, while gonads, membranes, and some lean tissues may offer a greater relative abundance of polar lipids [74,116,117]. High PUFA content increases susceptibility to oxidation, which can reduce nutritional quality and generate rancid odors and flavors. Freshwater fish lipid extracts should be evaluated using primary and secondary oxidation markers rather than fatty acid composition alone [84]. Exposure to oxygen, light, heat, pro-oxidative metals, lipoxygenase activity, and repeated freeze–thaw cycles should be minimized during extraction, storage, and food incorporation [118]. Refining may remove contaminants and undesirable odors but may also reduce phospholipids, tocopherols, pigments, and other minor constituents [119]. Microencapsulation, emulsification, liposomal delivery, and oleogelation may improve dispersibility, oxidative protection, and sensory compatibility. Nevertheless, the effectiveness of these approaches depends on wall material, interfacial composition, particle size, oxygen permeability, and the properties of the final food matrix [120,121]. Evidence obtained for marine fish oils cannot automatically be transferred to carp-derived oil because freshwater lipids may differ in n-6 content, phospholipid composition, pigments, oxidation kinetics, and flavor-active compounds. Direct food applications remain limited. Recent incorporation of common carp meat into breadsticks demonstrates that carp-derived material can increase the nutritional value of a cereal-based product, although such whole-tissue enrichment differs from adding a purified lipid fraction [101]. Future studies should assess standardized carp lipid ingredients in beverages, dairy analogues, bakery products, meat products, and emulsified foods, including their oxidative stability, sensory thresholds, digestion, bioaccessibility, and consumer acceptance.

#### 3.2.5. Squalene

When mentioning fish lipids, it is necessary to mention squalene (SQ). It is an isoprenoid lipid (a triterpenoid hydrocarbon composed of 30 carbon atoms) [90] with a similar structure to β-carotene [122]. Squalene is an important bioactive substance found in fish. It is an antioxidant that plays a crucial role in fat metabolism. Its antioxidant activity is associated with the scavenging of free radicals, especially reactive oxygen species (ROS). Squalene is a key precursor in the biosynthesis of fat-soluble vitamins, cholesterol, and steroid hormones. The richest natural and dietary sources of squalene are the livers of deep-sea sharks. Their concentration of pure squalene ranges from 60% to 85%. In plant products, squalene is found in amaranth seeds (6–8%) and olive oil (0.7%) [123,124]. It has been detected in muscle and visceral fats of several freshwater species [125]. Reported concentrations ranged from 98.0 to 1536.8 mg/kg of muscle fat and from 70.1 to 1803.8 mg/kg of visceral fat. The highest values were observed in rudd (*Scardinius erythrophthalmus*), with 1536.8 mg/kg in muscle fat and 1803.8 mg/kg in visceral fat. Among commercially important species, muscle fat concentrations were 386.6 mg/kg in common carp, 216.5 mg/kg in silver carp, 217.4 mg/kg in grass carp, 226.4 mg/kg in brown trout, and 354.4 mg/kg in wels catfish [124]. The average squalene content in commercially important freshwater fish, such as common carp, brown trout, grass carp, and silver carp, ranges from 176 to 386 mg/kg of muscle fat. Squalene as a dietary supplement in the feed of freshwater fish (rainbow trout, Siberian sturgeon, Eurasian perch) increases the content of polyunsaturated fatty acids (PUFA) in their tissues [126]. In the human body, squalene strengthens immunity and exhibits hypolipidemic [127], antioxidant, anticancer [128], and anti-inflammatory properties [123]. It also prevents skin aging and wrinkle formation [124]. Therefore, it is widely used in the food and pharmaceutical industries. Squalene, one of the best natural emollients, effectively absorbed into the skin, restoring its natural elasticity and firmness. It is an essential ingredient in cosmetics such as moisturizing creams, hair care products, lip care products, and nail care products. Properties such as odorlessness and colorlessness, ease of spreading, non-greasy consistency, rapid absorption through the skin, and antibacterial properties are additional advantages of squalene as an ingredient in cosmetic products [129]. Squalene is also used to treat eczema [130]. The human body naturally produces squalene, but the amounts are so small that it is worth supplementing it. It is assumed that 60–85% of squalene from the diet can be absorbed; therefore, it is recommended to take its exogenous form at a dose of approximately 500 mg/day [128]. In the human body, squalene is synthesized by the liver and secreted in large quantities by the sebaceous glands (sebum). In skin lipids, it constitutes approximately 500 µg/g and in adipose tissue 300 µg/g, whereas in organs where its biosynthesis occurs, the concentration is much lower–75 µg/g in the liver [122]. Due to its antibacterial properties, squalene inhibits the growth of pathogenic bacteria, including *Staphylococcus aureus*, stops food spoilage, and improves food quality [131,132]. The food industry is increasingly exploring the inclusion of squalene in various products (including bread and sweet bakery products) to improve their functional properties and enrich them with this unique ingredient [130]. In the form of tablets or capsules, it is also used in the diet as a supplement to improve health and well-being and as an adjuvant in vaccines. Squalene in the form of lyophilized powder and microcapsules can be incorporated into smoothies, juices, and baked goods to enhance their health-promoting properties. When used in food, the compound limits the oxidation of oxygen-sensitive ingredients, thus helping to preserve their high nutritional value, sensory properties, and shelf life.

### 3.3. Polysaccharides and Related Amino-Sugar Compounds

Polysaccharides obtained from freshwater fish constitute a smaller, less-studied group of bioactive components than proteins and lipids (Table 3). However, their importance is justified by their structural and biological functions, occurrence in processing waste, and potential use as nutraceutical or technological ingredients [133,134]. Additionally, they provide greater stability during processing and storage and contribute to improved texture and health benefits [135]. The most important fish-derived polysaccharides are glycosaminoglycans, particularly chondroitin sulfate, dermatan sulfate, heparan sulfate, and hyaluronic acid. These linear polysaccharides occur naturally in cartilage, notochord, connective tissue, skin, periosseous tissues, eyes, and other extracellular matrices.

Fish-derived polysaccharides should not be considered a uniform ingredient category. Chondroitin sulfate is primarily investigated as a nutraceutical compound, whereas high-molecular-weight hyaluronic acid can modify hydration, viscosity, and water retention [135]. Chitosan, when produced from a confirmed chitin-containing source, has antimicrobial, film-forming, flocculating, and coating properties [136]. Glucosamine differs fundamentally from these compounds because it is an amino-sugar monomer rather than a polysaccharide and is obtained through hydrolysis of chitin, chitosan, or other glucosamine-containing substrates [137]. Their production methods, functional properties, effective concentrations, stability, and regulatory requirements must therefore be evaluated separately.

The practical relevance of these compounds also depends on their tissue distribution. Cartilage-rich heads, backbones, fins, and notochords are potential sources of sulfated glycosaminoglycans, while the vitreous humour of the eye can provide hyaluronic acid [138,139]. Fish scales have been investigated as possible sources of chitin, chitosan, and glucosamine; however, this evidence remains less established than the recovery of chitin from crustacean exoskeletons [140,141]. Common carp is of particular interest because large quantities of scales, heads, bones, skin, and viscera are generated during processing. Nevertheless, direct studies demonstrating food-grade recovery and application of polysaccharides from common carp remain scarce.

**Table 3:** Representative polysaccharides and related amino-sugar compounds recovered from common carp and other freshwater fish.

Species and Source Material	Recovered Compound	Extraction or Processing Approach	Principal Finding	Potential Food Relevance and Major Limitation	Ref.
Rohu (*Labeo rohita*) and red-bellied pacu (*Piaractus brachypomus*) heads	Chondroitin/dermatan sulfate and heparan sulfate-rich GAG fractions	Proteolytic tissue digestion, polysaccharide isolation, fractionation, and disaccharide characterization	Species-dependent GAG composition and osteogenic activity were reported	Demonstrates recovery from freshwater fish by-products; no direct food-matrix, sensory, or bioavailability data	[142]
Nile tilapia (*Oreochromis niloticus*) processing residues	Chondroitin sulfate-rich fraction	Proteolysis, precipitation, purification, and structural analysis	Strong intracellular antioxidant activity was reported	Potential nutraceutical ingredient; evidence remains in vitro	[143]
Chinese sturgeon (*Acipenser sinensis*) head cartilage	Chondroitin sulfate and aggrecan-associated GAGs	Cartilage extraction and comprehensive structural characterization	Tissue-specific CS structures were identified	Relevant to ingredient standardization; no food application was tested	[144]
Chinese sturgeon notochord and backbone	Chondroitin sulfate	Extraction, purification, and comparative structural analysis	Sulfation patterns and biological activities differed between tissues	Demonstrates that tissue origin affects function; application evidence remains preclinical	[144]
Russian sturgeon (*Acipenser gueldenstaedtii*) notochord	Chondroitin sulfate and type II collagen	Sequential extraction and formation of hybrid fibrils	Combined recovery of polysaccharide and collagen fractions was demonstrated	Relevant to biorefinery processing; study focused on biomaterial rather than food use	[145,146]
Nile tilapia eyeballs	Hyaluronic acid	Tissue homogenization, enzymatic treatment, precipitation, and purification	Hyaluronic acid with defined structural characteristics was obtained	High-value source with possible rheological applications; food-grade safety and functionality were not established	[147]
Rohu scales	Chitin- and chitosan-like fractions	Acid demineralization, alkaline deproteinization, and deacetylation	FTIR and physicochemical analyses were consistent with chitin/chitosan	Preliminary evidence; requires stronger structural confirmation and food-grade purity assessment	[148]
Nile tilapia scales	Chitin and chitosan	Chemical demineralization, deproteinization, and deacetylation	The recovered chitosan reduced apple juice turbidity	Rare direct food application; toxicological, allergenicity, and regulatory validation remain necessary	[149]
Common carp scales	Chitin- and chitosan-like fractions	Chemical demineralization, deproteinization, decolourization, and deacetylation	Low ash content and a degree of deacetylation of approximately 57–63% were reported	Carp-specific evidence; degree of deacetylation and limited analytical validation restrict conclusions regarding food use	[150]

CS—chondroitin sulfate; GAGs—glycosaminoglycans; FTIR—Fourier-transform infrared spectroscopy.

#### 3.3.1. Chondroitin Sulfate and Other Sulfated Glycosaminoglycans

In freshwater fish, polysaccharides are represented almost exclusively by glycosaminoglycans (GAGs), i.e., linear heteropolysaccharides occurring naturally in the connective tissue of fish [141,142]. The best characterized GAGs obtained from freshwater fish are chondroitin sulfate (CS), often co-occurring with dermatan sulfate (DS), and hyaluronic acid (HA) [151]. Their presence and properties have been described primarily in species commonly farmed in freshwater aquaculture, such as tilapia (*Oreochromis* spp.) and sturgeon (*Acipenser* spp.), which further increases their application potential in terms of raw material availability and process standardization [147,152].

Freshwater fish, like marine fish, are a valuable source of chondroitin, which occurs primarily in their cartilage, skin, fins, bones, and connective tissues. They are a suitable source because they are cheap and readily available. Pharmaceuticals, cosmetics, and food are just some of the industries that use chondroitin. Both freshwater and marine fish provide alternative and safer sources of chondroitin than terrestrial animals (chickens, cows, pigs) [153]. It most commonly occurs as chondroitin sulfate (CS), composed of D-glucuronic acid and N-acetyl-D-galactosamine linked by a β-1,3-glycosidic bond [154,155]. Scientific studies have shown that chondroitin isolated from fish waste (carp, trout, tilapia) exhibits high purity and biological activity. It is used in supplements supporting joint health, particularly in the treatment of degenerative joint disease, as well as in cartilage and bone regeneration [156]. It exhibits anti-inflammatory effects, inhibits the release of inflammatory mediators, and supports tissue repair processes. It has strong antioxidant properties, reducing free radicals and supporting cell protection against oxidative stress [143]. The bioavailability of orally ingested chondroitin sulfate depends on molecular weight, sulfation pattern, gastrointestinal degradation, and interactions with the food matrix. Freshwater fish are increasingly being used as a source of chondroitin due to its availability, low cost, and high quality [157], but most published studies do not compare fish-derived preparations with commercial reference materials under standardized conditions.

Studies in sturgeon have shown that cartilage in the head, spine, and fins contains significant amounts of chondroitin sulphate, with its disaccharide structure and degree of sulfation depending on tissue location. These differences have direct functional significance, as they influence both the rheological properties and the potential biological activity of chondroitin sulphate, as documented in structural and biological analyses of sturgeon cartilage extracts [144,145,146].

From a food technology perspective, chondroitin sulfate is highly hydrophilic and can interact with water and proteins. However, at the concentrations typically considered for nutraceutical use, its contribution to viscosity or gel formation is likely to be smaller than that of conventional food hydrocolloids. Its principal relevance to food enrichment is therefore currently nutritional or nutraceutical rather than structural [157]. Application studies should determine whether the compound remains stable during pasteurization, fermentation, drying, and storage and whether it affects flavor, turbidity, color, or protein aggregation.

#### 3.3.2. Hyaluronic Acid

A unique component of fish vitreous humor is hyaluronic acid (HA), which has significant biomedical importance. In addition to its use in pharmaceuticals and its wide use in cosmetics, the compound is attracting increasing interest in the food industry. The Ministry of Food and Drug Safety has officially approved hyaluronic acid as a food additive. Currently, hyaluronic acid is used as a fortifying and quality-improving substance in European Union countries, the USA, Canada, and China [158].

Hyaluronic acid consists of the disaccharide units N-acetyl-D-glucosamine (GlcNAc) and D-glucuronic acid (GlcA), linked by alternating β (1 → 3) and β (1 → 4) glycosidic bonds. It also occurs naturally in the human body. It is a component of the extracellular matrix (ECM) of connective tissues, skin (50%), cartilage, joints, the vitreous humor, and the umbilical cord. Hyaluronic acid, depending on its molecular weight (HMW up to 1 MDa (high), MMW from 500 to 700 kDa (medium), LMW from 50 to 150 kDa (low), can perform various biological functions [159]. At high molecular weight (HMW), it enhances the differentiation of human monocytes into fibroblasts, while at low molecular weight (LMW) it has the opposite effect [160].

The anti-inflammatory and anti-angiogenic properties of hyaluronic acid are attributed to HMW. In this form, it can reduce lung inflammation, arthritis, and regenerate damaged skin. LMW hyaluronic acid exhibits a pro-inflammatory effect and can enhance the production of growth factors, which leads to increased cell proliferation and tissue regeneration. This form of hyaluronic acid (LMW) may also have angiogenic effects and stimulate the immune system [161]. Given the growing demand for hyaluronic acid not only in medicine and cosmetics, but also in food, the use of raw materials derived from fish rather than land animals is gaining increasing popularity due to safety, cost, and production considerations [162]. A good and effective solution is obtaining hyaluronic acid from the eyeballs of Nile tilapia (*Oreochromis niloticus*) with a high purity of 99.5% [147]. In marine fish, hyaluronic acid obtained from the vitreous humor and eyeballs of bluefin tuna also demonstrates high purity, with a significant content of uronic acid of 55.8% and a molecular weight of 880 kDa. Hyaluronic acid from aquatic sources has many beneficial health-promoting properties: It has anti-inflammatory and analgesic properties, and regenerates damaged tissues, cartilage, and joints [163]. It effectively heals wounds and prevents scarring. It is biocompatible, which means it is well tolerated by living tissues and does not cause allergic or adverse reactions in medical applications. It plays a key role in regulating tissue hydration and water transport, maintaining the elasticity and viscosity of connective tissues [164]. In addition to its health benefits, hyaluronic acid has many benefits in modifying functional food products. It has the ability to bind large amounts of water, making it a very effective additive in improving the texture and consistency of products. It is used in food as an emulsifier, thickener, and stabilizer. It increases product shelf life and enhances flavor and appearance. It can also be used as a sweet flavor enhancer in low-sugar diet products [129]. Due to its health-promoting properties, it also plays a role in improving product functionality, supporting skin health, joint health, and overall well-being. It is added to juices, yogurts, and confectionery products, including cakes, muffins, and candies [128].

#### 3.3.3. Chitin, Chitosan, and Glucosamine from Freshwater Fish Scales

Fish scale waste is a promising raw material for glucosamine production. This is confirmed by the latest research by Nurfaidah et al. (2025) conducted on scales obtained from Nile tilapia (*Oreochromis niloticus*) during gelatin production [30]. Produced by hydrolysis of chitin, glucosamine is free of heavy metals (As, Cd, Pb), but its purity index does not yet meet the standards specified by EFSA (2010) (LoD (after drying) ˂ 1% and Lol (after roasting) ˂ 0.1%) [165]. The manufacture of chitosan and glucosamine from scales requires careful optimization of deproteinization and demineralization. Demineralization is commonly conducted under acidic conditions to remove calcium phosphate and other mineral components, whereas deproteinization uses alkaline treatment, proteolytic enzymes, or microbial fermentation. Excessive acid exposure can hydrolyze glycosidic bonds and lower polymer molecular weight, while severe alkaline treatment can cause uncontrolled deacetylation, chain degradation, discoloration, and formation of poorly characterized reaction products. Insufficient treatment, by contrast, may leave residual minerals and fish proteins [166,167]. The LoD (6.01%) and LoQ (1.27%) limits obtained by Nurfaidah et al. (2025) exceed the established limits, indicating that the resulting product does not yet meet the requirements for food use and is currently more suitable for technical purposes. Therefore, further research is necessary to optimize the process [30]. Despite these limitations, fish waste such as scales remains a promising source of chitin and glucosamine, key compounds for the processing industry, with a wide range of applications. Their use also helps reduce pressure on the environment by reducing the amount of fish waste. Chemically, glucosamine is an amino sugar (2-amino-2-deoxy-β-D-glucopyranose) and is part of the structure of two polysaccharides, chitosan and chitin [168]. Glucosamine, in the form of hydrochloride, N-acetyl-D-glucosamine, or sulfate, is widely used as a dietary supplement. It supports joint function, including synovial fluid synthesis and cartilage regeneration. It is a fundamental building block for the biosynthesis of various compounds necessary for the formation and repair of cartilage tissue, such as glycolipids and glycoproteins, including proteoglycans [169].

A recent study reported the isolation of chitin and chitosan from common carp scales, but the estimated degree of deacetylation was approximately 57–63%. These values are relatively low for many applications conventionally assigned to chitosan and illustrate that chemical identification alone does not guarantee the functional or food-grade quality of the product [150]. Carp scales remain a potentially valuable processing side stream, but stronger analytical confirmation and direct food-safety studies are required before they can be considered a reliable source of chitosan or glucosamine.

#### 3.3.4. Functional Properties, Safety, and Application Limitations

From a food technology perspective, structure is crucial. The degree of sulfation, the distribution of sulphate positions, and molecular weight determine not only the potential biological activity (e.g., antioxidant or inflammation-modulating properties) but also the behavior of these polysaccharides in food matrices. Studies on GAGs isolated from freshwater fish processing waste have shown that even small differences in the disaccharide composition of CS/DS can result in distinct physicochemical properties, which are important for their use as food fortification ingredients [142,143,170].

In the context of food fortification, glycosaminoglycans from freshwater fish can serve a dual purpose. On the one hand, they exhibit biological activity, most often described as antioxidant potential or supporting connective tissue metabolism, which may justify their presence in functional foods [142]. On the other hand, as hydrophilic polysaccharides, they influence the product’s technological properties, including viscosity, water-binding capacity, and structural stability. Due to its strong hydrophilic properties, hyaluronic acid can improve the mouthfeel of semi-liquid products. At the same time, chondroitin sulfate can act as a structural support component in protein systems, although its hydrogel effect is weaker than that of classic hydrocolloids [171]. At appropriate levels, polysaccharides are usually taste-neutral [172], although excessive use—especially chitosan—may affect mouthfeel or cause cloudiness, requiring careful formulation [157].

The stability of GAGs during food processing and storage remains a significant issue. Available studies indicate that both CS and HA are sensitive to prolonged heating and extreme pH values, leading to depolymerization and loss of desired functional properties [171,173,174]. In the context of fortified foods, this means selecting technological processes that minimize polysaccharide degradation, such as mild pasteurization conditions or limited exposure to high temperatures. At the same time, it should be emphasized that potential negative sensory impacts typically result not from the polysaccharide itself, but from insufficient purification of extracts and the presence of residual lipids or fish proteins [142].

Another example of a polysaccharide used in food processing, also sourced from freshwater fish, is chitosan. Through partial or complete deacetylation, chitin is converted into chitosan, a cationic polysaccharide with increased solubility and functional versatility [175,176]. From the perspective of food fortification, chitosan is of particular importance due to its antimicrobial activity [177,178,179], film-forming and coating properties (enabling its use in edible coatings and active packaging to extend shelf life) [180,181], and lipid-binding capacity [182]. Chitosan is often obtained from crustaceans, but also directly from freshwater fish, in particular from scales. Kumari and Rath (2014) described the extraction and characterization of chitin and chitosan from the scales of rohu *Labeo rohita*, a commonly farmed freshwater fish, using demineralization and deproteinization steps [142]. The resulting chitosan exhibited characteristic FT-IR bands and a degree of deacetylation comparable to that of reference materials, confirming the feasibility of obtaining a functional polysaccharide from mineralized fish tissue. Freshwater fish scales may represent an alternative, although still poorly understood, source of chitosan.

Polysaccharides used for food fortification must meet stringent safety requirements and legal regulations. Key issues include allergenicity, particularly for chitin- and chitosan-based materials, purity, and control of contaminants, including heavy metals and residual proteins [183].

Despite the growing research publications, the use of glycosaminoglycans from freshwater fish in food remains underdeveloped. There is a lack of application studies in food matrices, with most available data focusing on isolation, chemical characterization, and in vitro testing. Knowledge about the influence of species, fish age, rearing system, and tissue type on GAG profile and functionality is also limited. Filling these research gaps is crucial for polysaccharides from freshwater fish to be recognized not only as interesting bioactive compounds but also as viable, safe, and functional ingredients for food fortification.

### 3.4. Pigments

The coloration of freshwater fish is shaped by the interaction of genetic, biochemical, environmental, and nutritional factors. The main pigments are melanin, carotenoids, pteridines, and guanine, the distribution and quantity of which determine the color of the fish’s flesh and skin [184]. The degree of pigmentation reflects the health and attractiveness of individuals and also influences the nutritional and culinary value of the meat. Pigments in freshwater fish are found in specialized chromatophore cells, which come in several types, each of which is responsible for a different color.

- Melanophores contain melanin, which gives black and brown shades. Melanin is produced by the enzymatic conversion of tyrosine by tyrosinase. Melanophores are responsible for dark colors, camouflage, and protection from UV radiation [185]. The distribution of melanin granules within melanophores influences the intensity of the fish’s surface color—they disperse, darkening the surface, and aggregate, brightening it [186].
- Xanthophores contain carotenoids and pteridines, which give yellow and orange colors. Carotenoids (astaxanthin, lutein, zeaxanthin) are obtained from the diet (algae, plankton, aquatic plants) and stored in muscles and skin. Xanthophores are responsible for intense colors, especially in fish eating foods rich in carotenoids [187].
- Erythrophores contain red pigments (carotenoids, pteridines) that give shades of red and orange [188].
- Iridophores contain guanine crystals that reflect light, creating an iridescent, silver, or blue effect. They are responsible for the metallic shine and color variation depending on the angle of incidence of light [189].
- Leucophores contain light-reflecting substances that give them a white color [190].
- Cyanophores are responsible for blue colors, less common in freshwater fish [191].

Pigments are distributed throughout the various layers of fish skin and muscles, and their quantity and proportions depend on the species, age, diet, environment, and genetic factors. Carotenoids, due to their wide range of colors, from yellow through orange to red, play an important role in fish coloration. Since fish cannot synthesize carotenoid pigments, dietary supplements are necessary to produce them [192]. Many fish species accumulate carotenoid pigments in their gonads, eggs, and skin. Salmonid fish have the ability to store astaxanthin in their muscles, giving them the intense orange color typical of this species. Astaxanthin is found not only in marine fish but also in freshwater fish. Tunaxanthin, responsible for the yellow color of fins and skin, is a common carotenoid pigment in perciform fish [193].

Unlike freshwater cyprinids, which can synthesize astaxanthin from zeaxanthin, perciformes and salmonids do not have this ability and must obtain this pigment directly from food [194]. Consumption of natural sources of carotenoids by wild fish and feed enriched with these pigments (astaxanthin, canthaxanthin) by farmed fish improves not only the quality of their meat but also its health properties [195]. Carotenoid levels in wild salmonids can range from 26 to 39 mg/kg in Sockeye salmon (*Oncorhynchus nerka*) to 8–9 mg/kg in Chinook salmon (*Oncorhynchus tshawytscha*), and in rainbow trout (*Oncorhynchus mykiss*) over 3 mg/kg [194]. This confirms that they are a good and valuable source of carotenoid pigments in the human diet [196]. From the point of view of consumer protection, it is better to feed fish with natural sources of carotenoids (green microalgae *Haematococcus pluvialis*, red yeast *Xanthophyllomyces dendrorhous*, bacteria *Paracoccus carotinifaciens*, krill oil and meal, red pepper) instead of their synthetic counterparts, which are not neutral to the human body [197]. Using natural astaxanthin in its esterified form increases its lipophilicity, bioavailability, and retention in fish muscle tissues. However, chemically synthesized astaxanthin (non-esterified form) is unstable and produces less intense coloration in fish [198].

Astaxanthin derived from natural sources (*algae*) has a higher antioxidant potential and pigmentation efficiency. Although synthetic astaxanthin remains the dominant compound in large-scale aquaculture due to its cost-effectiveness and reproducible pigmentation, natural astaxanthin is increasingly preferred in production systems due to its higher bioactivity, color intensity, and favorable consumer perception [199]. Consuming salmonid fish, including rainbow trout, rich in carotenoids, offers numerous health benefits. These compounds have antioxidant properties that are associated with the quenching of singlet oxygen and the scavenging of free radicals. The antioxidant properties of dietary carotenoids also contribute to their anticancer and anti-inflammatory effects. In addition to these properties, some carotenoids reduce obesity and counteract diabetes [200]. The role of carotenoids in preventing cardiovascular disease, including heart attack and stroke, is invaluable. Carotenoids also support the function of B and T lymphocytes, macrophages, and other leukocytes involved in the body’s defense mechanisms. Their properties are also utilized in the production of cosmetics and sunscreen products [201].

The use of freshwater fish-derived carotenoids in fortified foods remains insufficiently investigated. Most available studies focus on feed pigmentation, fish growth, or physiological responses rather than on the recovery of pigments and their incorporation into another food matrix. Furthermore, carotenoid concentrations in freshwater fish muscle are generally lower than those obtained from microalgae, crustacean by-products, or dedicated microbial production systems [202,203,204,205]. Fish-derived carotenoid fractions should therefore be considered possible co-products of tissue or lipid valorization rather than established high-yield alternatives to existing commercial pigment sources.

Myoglobin is an oxygen-binding heme protein and an important determinant of muscle color. Its significance in food systems results not only from its red color but also from its participation in oxidative reactions. Oxidation of ferrous myoglobin to ferric metmyoglobin causes brown or grey discoloration, while heme iron can promote lipid and protein oxidation during refrigerated or frozen storage [198]. The technological relevance of myoglobin therefore concerns color stability, rancidity development, protein functionality, and consumer perception rather than its use as an isolated fortifying compound. Its concentration ranges from 1.8 to 2.8 mg/g of muscle, depending on the species, fish activity, and environmental conditions. In common carp, myoglobin is distributed unevenly among the muscle fiber types, with greater immunohistochemical abundance in red oxidative fibers than in intermediate and white fibers [206]. Accordingly, ingredients containing a larger proportion of dark lateral muscle may have a darker color and greater pro-oxidative potential than ingredients produced predominantly from white dorsal muscle. In rainbow trout, myoglobin expression is highest in the heart and slow skeletal muscle, although the protein has also been detected in several non-muscle tissues [207]. Channel catfish fillet color is influenced by myoglobin, residual hemoglobin, and their oxidation products. Bleeding before processing can reduce heme-associated discoloration and limit interactions between myoglobin, lipid oxidation, and myofibrillar protein oxidation during repeated freeze–thaw cycles [208,209]. The color of the flesh of freshwater fish is considered an important indicator of quality with which consumers associate freshness and the desire to purchase it.

### 3.5. Vitamins

The fat-soluble vitamins in freshwater fish are all-trans-retinol, cholecalciferol, and α-tocopherol most often. Vitamin D in native fish tissue is represented predominantly by vitamin D_3_, or cholecalciferol. Some fish tissues also contain 25-hydroxyvitamin D_3_, which possesses biological vitamin D activity but is usually present at lower concentrations than cholecalciferol. Their concentrations vary substantially among species, tissues, diets, production systems, seasons, and analytical methods. Moreover, vitamins may be lost during extraction, refining, heating, drying, and storage [210,211].

Freshwater fish should not be classified collectively as rich sources of fat-soluble vitamins. Species-specific studies demonstrate considerable variability. In one analysis of common carp from the Danube River, the edible tissue contained 30.8 ± 3.4 µg/100 g retinol, 14.8 ± 1.0 µg/100 g cholecalciferol, and 2764.5 ± 44.0 µg/100 g α-tocopherol [212]. However, these findings represent fish from a specific habitat and sampling period and should not be generalized to all common carp populations.

Likewise, reported vitamin D_3_ concentrations in freshwater fish are variable. One investigation found an average vitamin D_3_ content of 9–44 μg/100 g in carp meat, demonstrating that it may contribute meaningfully to dietary vitamin D intake but may not consistently contain the high values commonly attributed to fish [213]. The vitamin D content of aquaculture products can be influenced by feed composition [211]. This creates an opportunity for controlled biofortification but also indicates that species identity alone is insufficient to predict vitamin content.

Retinol and α-tocopherol are associated mainly with lipid-containing tissues and may be partly retained in fish oils or minimally refined lipid extracts. α-Tocopherol is technologically relevant because it contributes to the protection of unsaturated lipids against oxidation. Nevertheless, its concentration may decrease during prolonged storage and intensive refining. Retinol is also sensitive to oxygen, light, and heat [214]. The actual nutritional contribution of a freshwater fish-derived ingredient must therefore be determined after processing and in the final product rather than inferred from the raw material.

Among the water-soluble vitamins, vitamin B_12_, niacin, vitamin B_6_, riboflavin, and thiamine may contribute to the nutritional value of freshwater fish muscle. Their relevance is greatest when whole muscle, mechanically separated meat, protein-rich powder, or minimally fractionated biomass is incorporated into food. Water-soluble vitamins are less likely to remain in purified oil fractions and may be partly lost with aqueous process streams or through leaching during cooking [214,215]. Consequently, the fortification value of a fish-derived ingredient depends strongly on its production route.

### 3.6. Minerals

Freshwater fish, including carp and trout, are a valuable source of minerals essential for the proper functioning of the human body. The micro- and macronutrients contained in fish are highly bioavailable, making them easily digestible. The mineral composition of fish depends on the species, habitat, diet, and age of the fish [216]. Feeding habits, migration patterns, and ecosystem conditions further influence the accumulation of trace elements, which contribute to varying ash content, even among fish coexisting in the same habitat [217]. Freshwater fish muscle generally provides potassium and phosphorus and contains smaller quantities of magnesium, iron, zinc, selenium, and other trace elements. Common carp fillet is particularly relevant as a source of potassium, whereas its contribution to calcium intake is limited when the bones are removed. In a recent tissue-specific analysis, potassium was the most abundant mineral in common carp fillet, reaching approximately 4005 mg/kg, while the calcium concentration in carp bones reached 116,463 mg/kg [205]. These results demonstrate that the mineral profile of the fillet cannot be used to characterize the nutritional potential of the entire processing biomass. Freshwater fish, including carp, are a rich source of potassium, which supports healthy blood pressure and heart function (Table 2). Magnesium is essential for the proper functioning of muscles and the nervous system, and participates in over 300 enzymatic reactions. Carp meat provides magnesium in healthy amounts. Calcium is crucial for bone and tooth structure, as well as for nerve conduction and blood clotting. Although freshwater fish are not as rich in calcium as dairy products, soups made from the carcasses of these fish or cutlets made from minced small fish (e.g., roach) can be a good alternative source of calcium in the human diet. It is assumed that bones, which constitute about 10–15% of the total biomass of fish, can serve as a source of minerals beneficial to health in the diet [93,218].

Fish bone powder, obtained by drying and grinding fish bones, has potentially beneficial health properties in the human diet. It is rich in essential minerals such as calcium (Ca), phosphorus (P), iron (Fe), and biofunctional collagen protein [219]. It is a highly effective food additive for pasta, cookies, and rice crisps, and also serves as a dietary supplement [220]. In addition to its high calcium and phosphorus content, powder obtained from freshwater fish bones is also characterized by good absorption by the human body [221]. Fish meat also contains iron at approximately 0.4–0.9 mg/100 g, which has higher bioavailability than iron from plant products. This nutrient is essential for hemoglobin production and the prevention of anemia. Zinc supports the immune system and participates in the synthesis of proteins and nucleic acids, as well as accelerating wound healing [222]. Carp and other freshwater fish (Table 4) contain zinc in amounts beneficial to health. Selenium is a powerful antioxidant that protects cells from oxidative stress and supports thyroid function [223]. Freshwater fish, including carp, can be a good source of selenium, although their content depends on the aquatic environment.

Common carp contains potassium, phosphorus, zinc, and iron at levels comparable to other freshwater and marine fish species (Table 5). The lower sodium and higher potassium concentrations in carp meat indicate its suitability in a diet to prevent cardiovascular disease, where the desired Na/K ratio should not exceed 1 [226].

Freshwater fish contain significantly less iodine than marine species. Common carp contain 1.5–3.0 μg/100g, and rainbow trout 11–13 μg/100 g. Differences in iodine content are primarily due to the natural habitat of the fish. Seawater contains iodine in much higher concentrations (50–60 μg/L) and even 100 μg/L, while freshwater ranges from 0.1 to 10.0 μg/L [227,228].

The use of mineral-rich carp by-products for food enrichment also requires comprehensive safety assessment. Bones and other mineralized tissues may accumulate lead, cadmium, mercury, arsenic, or other environmental contaminants. Their concentrations depend on the origin and age of the fish, water quality, feeding conditions, and anatomical tissue. Food-grade production must consequently include batch-specific contaminant analysis, microbiological control, traceability, and verification of compliance with legal limits [213,229,230,231].

## 4. Extraction and Processing Technologies Used in Food Technology

Using freshwater fish as a source of bioactive ingredients for food enrichment requires the use of extraction and processing technologies that ensure high recovery efficiency, retention of biological activity, and final product safety. Unlike processes developed solely for analytical or pharmaceutical purposes, technologies used in the food industry must meet additional requirements regarding acceptable solvents, process scalability, and minimizing negative impacts on the product’s sensory properties [232,233]. In the case of fish raw materials, the effective use of processing by-products, such as skins, bones, scales, and viscera, is also particularly important. These are valuable sources of proteins, lipids, polysaccharides, and other bioactive compounds.

### 4.1. Conventional Extraction Methods

Conventional extraction methods remain the basis for industrially extracting bioactive compounds from raw fish. They primarily include mechanical processes, heat treatment, and extraction using food-grade solvents [9,234,235].

Mechanical separation is one of the simplest and most commonly used stages of fish processing [236]. This process involves grinding, homogenizing, and separating muscle, fat, and bone fractions using presses, separators, or centrifuges [237]. This allows for the initial concentration of the raw material containing specific bioactive compounds. These methods are particularly useful for recovering muscle proteins and lipids from raw materials such as mechanically separated meat, filleting waste, and fatty tissue. Mechanical separation of fish meat from bones (MSFP—mechanically separated fishery product) allows for obtaining a raw material that can be used as a starting material for further extraction of bioactive components [236,238].

Heat treatment is commonly used to recover proteins and lipids from fish raw materials. Heating the raw material denaturates the proteins and coagulates the protein fraction, facilitating the separation of lipids and other soluble components. This process is commonly used in the production of protein concentrates, protein hydrolysates, and fish oils. However, temperature and process time must be controlled, as excessive heat treatment can lead to the degradation of sensitive bioactive compounds such as peptides and polyunsaturated fatty acids [239,240].

Another commonly used method is solvent extraction, primarily used to obtain lipids and pigments from fish raw materials [237]. In food technology, food solvents such as ethanol or isopropanol are mainly used [241,242]. This method allows for the selective extraction of lipid fractions containing omega-3 fatty acids. This extraction is relatively simple technologically, but requires thorough removal of residual solvents and control of process conditions to prevent lipid oxidation [241].

A similar approach is also used in the extraction of some polysaccharides and glycosaminoglycans, although in these cases, solvent extraction is often combined with enzymatic hydrolysis and purification steps [243].

### 4.2. Enzymatic and Fermentation Processes

Enzymatic processes are one of the most important technologies used in the extraction of bioactive components from freshwater fish, particularly proteins, peptides, and polysaccharides. Enzymatic hydrolysis involves the use of proteolytic enzymes to degrade structural proteins into shorter peptides with potential biological activity [234,244]. This method is widely used in the processing of skin, bones, and fish meat, as it allows for simultaneous increased protein recovery and improved bioavailability [245]. This process involves the degradation of proteins by proteolytic enzymes such as pepsin or trypsin [246,247].

Enzymatic hydrolysis is also used in the isolation of polysaccharides, such as glycosaminoglycans, where proteolytic enzymes remove protein fractions from tissues rich in extracellular matrix. As a result, it is possible to obtain high-purity fractions of chondroitin sulfate or hyaluronic acid [136,248].

In recent years, microbial fermentation has also gained increasing interest as a method for processing fish raw materials [249]. During fermentation, microorganisms produce enzymes that degrade proteins and polysaccharides into more easily digestible forms and can generate new bioactive compounds. Fermentation also contributes to improving the sensory properties of fish raw materials by reducing undesirable aroma and microbiologically stabilizing the product [250].

### 4.3. New and Ecological Processing Technologies

In recent years, there has been a growing interest in extraction technologies known as “green” or ecological, which enable more efficient and environmentally friendly extraction of bioactive compounds from biological raw materials [251].

Low-frequency power ultrasound, generally within the range of 20–40 kHz, can be used as a pretreatment or processing aid during the recovery of proteins, collagen, hydrolysates, and lipids from fish tissues [252]. The use of ultrasound in the processing of fish raw materials significantly increases the extraction efficiency of many bioactive compounds, including proteins, peptides, lipids, pigments, and polysaccharides. In the case of the protein fraction, ultrasound can facilitate the breakdown of muscle structures and increase the availability of substrate for proteolytic enzymes. Studies on protein extraction from fish raw materials indicate that the use of sonication can improve both protein recovery efficiency and its functional properties, such as emulsification and water binding capacity [253]. In golden carp (*Probarbus jullieni*) skin, ultrasound treatment at 20 kHz, amplitudes of 20–80%, and pretreatment times of 10–30 min increased the recovery of acid- and pepsin-soluble collagen. Treatment at 80% amplitude for 30 min increased the yield of acid-soluble collagen to 81.53%, compared with 51.90% for conventional extraction. The combination of the same ultrasound conditions with 1% pepsin increased pepsin-soluble collagen recovery to 94.88%, while the principal type I collagen structure remained detectable [254]. These findings indicate that ultrasound may reduce mass-transfer limitations without necessarily destroying the collagen triple helix when process severity is adequately controlled.

Short-duration ultrasound treatment may also modify the functionality of previously produced protein hydrolysates. Fish protein hydrolysates treated at 20 kHz and 300, 450, or 600 W for 10 min exhibited increased solubility and changes in color and degree of hydrolysis. Nevertheless, treatments at 450 and 600 W increased carbonyl formation, and the highest acoustic intensities did not consistently improve antioxidant or dipeptidyl peptidase-IV inhibitory activity [255]. Thus, greater ultrasound power should not be assumed to produce a more functional ingredient.

A significant advantage of ultrasound-assisted extraction is its low-temperature nature. Unlike many conventional extraction methods, sonication can be conducted at temperatures close to room temperature, which limits the degradation of heat-sensitive compounds. This makes this method particularly useful for obtaining thermostable bioactive components, such as certain peptides, enzymes, and polyunsaturated fatty acids [256].

Another method for extracting fish raw materials is high-pressure processing (HPP). This technology involves subjecting biological material to high hydrostatic pressure, typically in the range of 100–800 MPa, in the presence of a pressure-transmitting medium, most often water. Unlike conventional thermal methods, this process takes place at a relatively low temperature, thus preserving many of the raw material’s sensory and nutritional properties [257,258]. The use of high pressure causes partial unfolding of protein structures and increases the availability of substrate for proteolytic enzymes, which can increase the efficiency of bioactive peptide production. In addition, pressure may promote the release of lipids and pigments from fish tissues by loosening the structure of cell membranes and protein–lipid aggregates [259]. The literature also indicates that HPP can improve the availability of bioactive components by modifying protein structure and increasing their susceptibility to enzymatic digestion. At the same time, in many cases, limited changes in the nutritional and sensory values of fish products are observed, which is one of the key advantages of high-pressure technology compared to traditional thermal processing [260].

Subcritical water extraction is an alternative to traditional organic solvents. This method involves the use of water at temperatures ranging from approximately 100 to 374 °C under elevated pressure, which maintains it in a liquid state. Under these conditions, the physicochemical properties of water change, making it an effective extraction medium for many bioactive compounds, including polysaccharides and some pigments [261]. Although there are still a few studies explicitly described regarding freshwater fish, the literature on fish waste and protein biomass indicates that this technology has significant potential for the recovery of protein fractions, peptides, and collagen components from fish-derived raw materials [262,263,264].

Supercritical carbon dioxide (SC-CO_2_) extraction is a technology for recovering lipid bioactive compounds from fish raw materials and is particularly important for freshwater fish, especially when the raw material is viscera, skin, roe, or other fat-rich byproducts. Its main advantage is the ability to conduct the process at moderate temperatures without leaving residual organic solvents, which is important for the isolation of thermolabile components such as omega-3 polyunsaturated fatty acids. Lisichkov et al. (2014) demonstrated that carp (*Cyprinus carpio*) viscera can be a viable source of omega-3 and omega-6 PUFA-rich fractions, and SC-CO_2_ is a suitable technique for their isolation [260]. Melgosa et al. (2020) study on extracts from sardine (*Sardina pilchardus*) waste demonstrates a model that is also very relevant for freshwater fish: first, recovery of the lipid fraction using the SC-CO_2_ method, followed by further use of the defatted biomass for the production of protein hydrolysates using subcritical water [264]. From a technological point of view, it is also important that SC-CO_2_ can act as a preparatory step before the recovery of other bioactive fractions. Removing lipids from viscera or other fatty byproducts of fish can improve subsequent extraction of proteins, peptides, or enzymes by reducing fat interference and improving the stability of subsequent unit operations [265,266]. Despite many advantages, this technology also has limitations. The most important are the high cost of pressure equipment, the need for appropriate raw material preparation, and yield variability depending on the species, tissue type, and material moisture content. In the case of freshwater fish roe, this shows that not every lipid matrix can be equally effectively extracted with supercritical CO_2_ without further optimization or the use of a cosolvent [267]. In turn, studies on carp and African catfish viscera indicate that the technology is most promising where fat-rich waste is processed and where fractions with significant added value for functional foods can be obtained [261,268].

### 4.4. Stabilization of Extracts

After extraction, a crucial step is stabilization and transformation of the obtained extracts into a form suitable for use in food products. Processes such as spray drying, freeze-drying, and microencapsulation are used for this purpose. Many compounds obtained from fish, such as polyunsaturated fatty acids, bioactive peptides, pigments, and certain polysaccharides, exhibit significant sensitivity to environmental factors, including oxygen, light, temperature, and humidity [269]. Therefore, developing appropriate stabilization methods is a key element of bioactive extract processing technology.

One of the most commonly used methods for stabilizing bioactive ingredients in the food industry is spray drying. This technology transforms liquid extracts into stable powders by atomizing the solution or emulsion in a stream of hot air [270]. In the case of bioactive ingredients, this process is often combined with microencapsulation, as the active substance is surrounded by a capsule wall material such as maltodextrin, whey proteins, modified starch, or vegetable gums. Microencapsulation significantly increases the stability of sensitive compounds, limiting their oxidation and degradation during storage [271]. This technique is particularly important for fish lipids containing polyunsaturated fatty acids, which are susceptible to oxidation and loss of nutritional value. Microencapsulation of fish oil through spray drying not only extends the shelf life of the product but also reduces undesirable aroma and flavor, which is important for products enriched with fish-derived ingredients [272,273]. In the case of protein hydrolysates and bioactive peptides, microencapsulation has the additional advantage of reducing undesirable sensory properties, such as bitter taste, which often accompanies products resulting from intensive protein hydrolysis. Encapsulation masks these characteristics while protecting the peptides from degradation during storage or further food processing [274].

An alternative method for stabilizing bioactive extracts is freeze-drying, which involves removing water from the frozen material by sublimating the ice under reduced pressure. This process preserves the structure and biological activity of many sensitive compounds, such as bioactive peptides and probiotic microorganisms, which can then be used as ingredients in functional foods [10,275].

### 4.5. Biorefinery Approach for Freshwater Fish Processing

**Figure 1:**
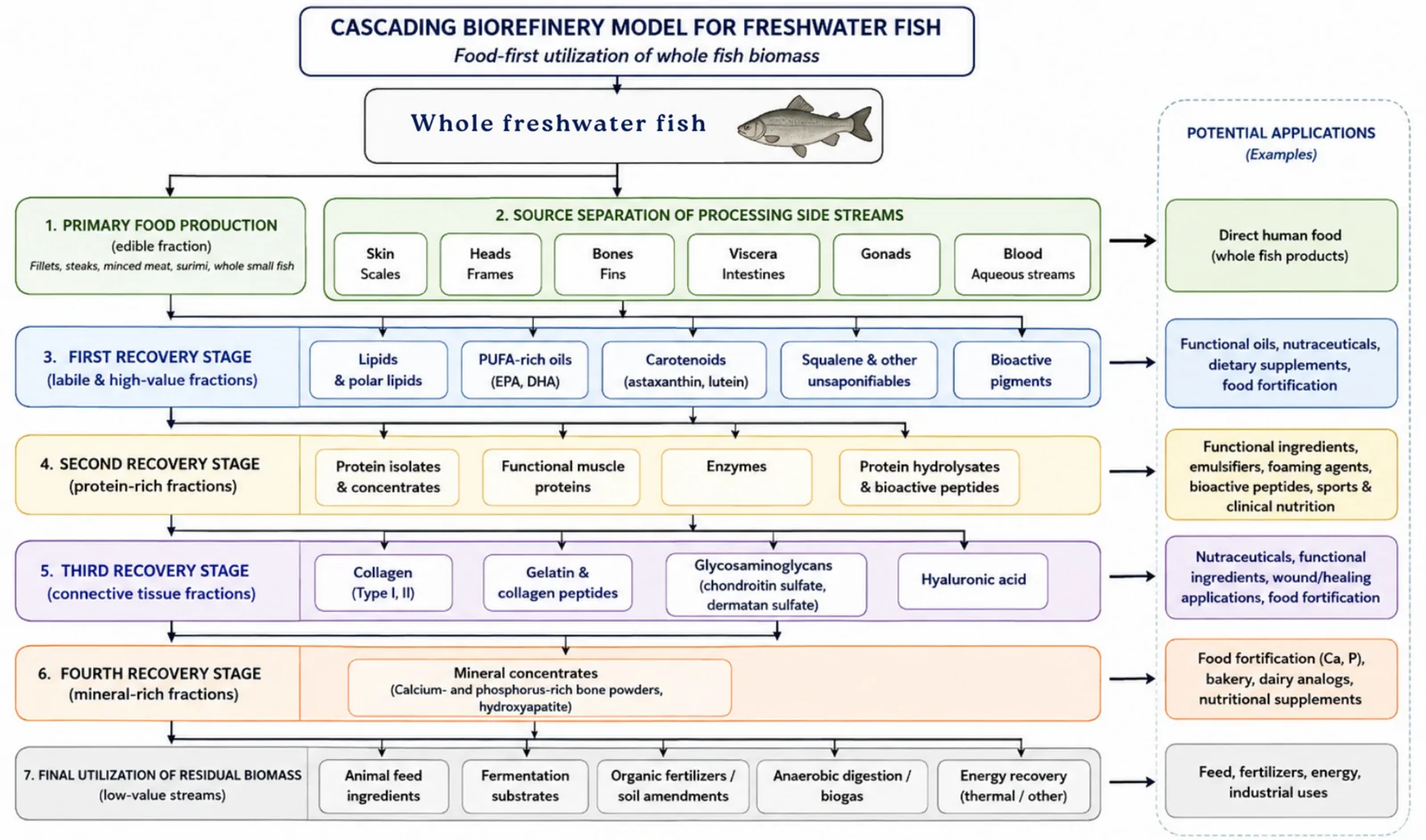
Food-first cascading valorization model for common carp and other freshwater fish processing biomass.

Biorefinery is defined in the literature as the sustainable processing of biomass into a range of bio-based products, including food, feed, chemicals, materials, and bioenergy. Figure 1 presents a food cascading valorization model for freshwater fish processing biomass. In the case of fish raw materials, this means moving away from a linear “disposal waste” model towards a model in which subsequent processing side streams become raw materials for the recovery of lipids, protein hydrolysates, collagen, glycosaminoglycans, minerals, and ultimately energy or fertilizers [276]. For fish and processing waste, this means using raw materials such as heads, skins, bones, scales, and viscera to produce bioactive components, including PUFA-rich lipids, protein hydrolysates, collagen, glycosaminoglycans, and minerals. The use of a biorefinery model allows for the simultaneous reduction in waste and increase in the economic efficiency of processing fish raw materials [277].

From a technological point of view, the key advantage of the biorefinery model is the ability to establish a recovery hierarchy, in which the most sensitive and valuable components are extracted first, and only then the remaining mass is sent for further processing. In practice, this means that lipids rich in long-chain n-3 fatty acids can be recovered from fish raw materials first, followed by protein and collagen fractions, then polysaccharide and mineral compounds, and the final residues can be subjected to fermentation, composting, or energy recovery [278].

In the context of fortified food production, the biorefinery recovery sequence must be subordinated to the food quality of the final fractions, rather than solely maximizing total mass recovery. This means designing the process so that the initial stages do not compromise the quality of the subsequently recovered fractions [277]. In a study by Fornaro et al. (2024), the recovery of omega-3 fractions from fish waste using supercritical CO_2_ was proposed, followed by further processing of the defatted biomass to recover collagen [279]. The authors clearly demonstrated that the advantage of this solution lies not only in the high recovery efficiency and good purity of the lipid fraction but also in preserving the integrity of the remaining biomass for subsequent protein use [280].

This principle is particularly valuable when designing biorefinery applications for freshwater fish. Raw materials such as skins and bones can be sources of collagen and gelatin, cartilage tissues (glycosaminoglycans), viscera and meat adjacent to the skeleton (protein hydrolysates), and fat fractions (PUFA-rich fat) [281]. However, if excessive heat treatment or aggressive chemical extraction is applied at the initial stage, irreversible degradation can occur not only of the target component but also of the fractions intended for subsequent recovery [279].

From a future research perspective, the greatest value will be derived from studies that combine classical extraction studies with a technoeconomic and environmental assessment of the entire system, rather than analyzing each fraction separately. The literature clearly demonstrates that the full value of fish biomass is only realized when the system is designed as a multi-product system, taking into account the quality of the final fractions, energy consumption, process water, enzyme costs, and the potential for commercialization of hydrolysates, lipids, and structural components [282]. This approach is particularly necessary for freshwater fish, for which coherent biorefinery models aimed at producing ingredients for food enrichment are still lacking.

## 5. Risk–Benefit Assessment and Safety of Freshwater Fish-Derived Bioactive Ingredients

Aquatic foods, including freshwater fish, provide many valuable nutrients, but they can also contain toxic, allergenic, and other compounds that reduce their quality and pose a health risk. It is worth emphasizing that fish meat is increasingly consumed in our diets, making assessment of its safety particularly important [283]. Carp meat can be considered a safe food product in terms of Hg, Pb, and Cd content, as the levels of these pollutants are lower than the maximum values specified in the FAO and European Commission guidelines. This is confirmed by the study by Skałecki et al. (2018), in which lead levels in the muscle tissue of the tested fish were lower than the maximum values recommended by the FAO/WHO and the European Commission for fish intended for human consumption (0.3 mg/kg) [93]. Carp meat also did not exceed the applicable limits for cadmium (0.05 mg/kg) and mercury (0.05 mg/kg) specified in EU regulations. These data are consistent with previous observations indicating that fish and fish products available on the Polish market have safe levels of heavy metals. According to Staszowska et al. (2013), the lead concentration in the muscle tissue of carp from Polish aquaculture was 0.043 mg/kg, confirming the low accumulation of this element in farmed species [283].

Heavy metals (mercury, cadmium, lead, arsenic, chromium, zinc, copper, nickel) enter the aquatic environment as a result of human activity (industry, fuel combustion, agriculture, sewage) and natural geological processes. In marine and freshwater environments, they can accumulate in sediments, plants, and aquatic organisms, including fish [284]. The highest concentrations are observed in marine fish, especially predatory fish that occupy the highest trophic levels (tuna, swordfish, shark, cod) [285]. In contrast, freshwater fish such as carp and trout have lower concentrations of heavy metals. In Baltic fish (mackerel, flounder, pike-perch, herring, salmon) studied by Ronda and Cieślik (2022), mercury concentrations were in the range of 0.08–0.13 mg/kg fresh weight, while the content of other metals (Cr, Cd, Ni, Zn) was at acceptable levels according to EFSA guidelines [286]. In fish, lead accumulates mainly in the liver, kidneys, spleen, and gills [287]. Poisoning by this metal in fish can occur at concentrations of 10–100 mg/L [288]. Due to its highly toxic nature, mercury is the third most toxic substance to the environment, after lead and arsenic, according to the EPA (United States Environmental Protection Agency) and ATSDR (Agency for Toxic Substances and Disease Registry) [289]. Exposure to heavy metals and other environmental pollutants that accumulate in fish can lead to some adverse health effects in humans, the nature of which depends on the dose, duration of exposure, and the individual health status of the exposed individuals [290,291,292,293].

The presence of biogenic amines in fresh fish and fishery products also poses a serious threat to food safety, as their increase is closely related to spoilage processes, microbial activity, and storage conditions [294]. In the natural physiology of fish, biogenic amines perform regulatory functions and are precursors of hormones, alkaloids, nucleotides, proteins, and aromatic compounds [295]. They are produced mainly as a result of decarboxylation of free amino acids with the participation of decarboxylase secreted by microorganisms (*Enterobacteriaceae*, *Lactobacillaceae* and *Pseudomonadaceae*) [296], as well as amination and transamination of aldehydes or ketones [297]. Biogenic amines found in freshwater fish are histamine (HIS) and tyramine (TYR) (monoamines) formed from the precursor amino acids histidine and tyrosine, then diamines include cadaverine (CAD), obtained from lysine; putrescine (PUT) from ornithine; and agmatine, or indirectly after hydrolysis of arginine [298]. Polyamines, including spermine (SPM) and spermidine (SPD), also belong to the biogenic amines, are produced by several metabolic pathways, in which arginine plays a key role. It can be hydrolyzed to ornithine or agmatine, which are key precursors in the biosynthesis of these compounds [299].

Fish are particularly susceptible to the accumulation of biogenic amines because they contain high levels of free amino acids, especially histamine precursors (histidine). They are highly unstable and spoil quickly. They are often subject to improper cooling or disruption of the cold chain, which promotes bacterial growth. The method of fish preparation also determines the amount of biogenic amines. For example, in the meat of carp, grinding resulted in a faster accumulation of putrescine, histamine, cadaverine, spermine, spermidine, tyramine, and tryptamine compared to whole muscle, most likely due to the exposure of a larger number of substrates (proteins, peptides, amino acids) to enzymes [300]. The increase in biogenic amines was also more intense in rainbow trout fillets than in ungutted fish [301]. Biogenic amines are not neutral to the human body. Their consumption can be associated not only with food poisoning but also with numerous allergic reactions. Histamine is the most abundant biogenic amine in fish, and therefore its content is an indicator for estimating its degradation and quality deterioration [302]. According to Muscarella et al. (2013), histamine content below 10 mg/kg indicates good quality fish, while 30–50 mg/kg indicates poor quality [303]. Consumption of 8–40 mg in a meal results in mild poisoning, and 40–100 mg in a meal results in moderate to severe poisoning [304]. Biogenic amines pose a potential threat to consumer health; therefore, preventing their formation and constant monitoring of raw fish is crucial. Primarily, low-temperature processing [305], the use of ionizing radiation [306], and the introduction of natural products that inhibit the formation of biogenic amines, including spices and extracts from safflower (*Carthamus tinctorius*) and bitter melon (*Momordica charantia*) [307]. The use of phenolic compounds (carnosic acid, luteolin, kaempferol) also reduces the formation of biogenic amines in fish [308]. Packaging fish raw materials in a modified atmosphere and vacuum inhibits the growth of microorganisms and reduces the level of biogenic amines. The introduction of H_2_ (molecular hydrogen) into modified atmosphere packaging, including particularly high levels of CO_2_ (46% and 60%), reduces the formation of biogenic amines in rainbow trout and horse mackerel stored for 15 days at 4 °C [309].

Fish, including freshwater fish, are a common cause of allergic reactions in humans. The incidence of fish allergy symptoms varies greatly between regions of the world and between children and adults [310]. It is assumed that the incidence of allergies is higher in countries with high fish consumption and developed fish processing industries, such as Norway, Sweden, Denmark, Spain, Portugal, Iceland, China, Peru, Chile, Japan, Russia, and the United States [311]. The main fish allergens are parvalbumins (PA), water-soluble muscle proteins with a molecular weight of 12 kDa, which are responsible for 95% of IgE-mediated sensitizations [312]. They belong to the family of EF- hand calcium-binding proteins (CBPs), which are characterized by a relatively conserved protein structure [313]. They are allergens with high cross-reactivity. Fish allergenicity is related to the presence of β-parvalbumins found in bony fish. The high structural stability of the calcium-saturated form provides relative resistance to cooking, even baking, and digestion in the gastrointestinal tract. Their IgE-binding capacity and allergenicity decrease after calcium ion depletion. Even if the protein is partially denatured, its fragments (epitopes) recognized by IgE antibodies in the allergic individual still retain the ability to trigger a reaction. Therefore, a clinically confirmed fish allergy occurs regardless of whether the product was consumed raw (sushi) or after cooking, baking, or frying. The risk of symptoms—from mild urticaria, through gastrointestinal symptoms, to life-threatening anaphylactic shock—remains high. Parvalbumin concentrations vary significantly between fish species, which partially explains why allergic reactions in patients may be more severe to some species and less severe to others. Therefore, the fundamental principle of dietary management of fish allergy remains complete elimination of fish, regardless of preparation method. Specific parvalbumins have been identified for specific freshwater fish species: Cyp c1 in the common carp (*Cyprinus carpio*), Onc m1 in the rainbow trout (*Oncorhynchus mykiss*), Sti l1 in the pike-perch (*Stizostedion lucioperca*), Ang a1 in the eel (*Anguilla anguila*), and Ict p1 in the channel catfish (*Ictalurus punctatus*). In marine fish, parvalbumin Sal s 1 is found in *Atlantic salmon* (*Salmo salar*); in Atlantic cod (*Gadus morhua*), Gad c1 and Gad m1, and in herring (*Clupea harengus*), Clu h1. In addition to parvalbumin, enolases, aldolases, and fish gelatin also exhibit allergenic properties. Patients’ allergy to fish can manifest through various routes: alimentary, respiratory, and skin contact. Typical allergic symptoms caused by fish include oral allergy syndrome, rhinitis, abdominal pain, diarrhea, urticaria, angioedema, asthma, and, in severe cases, anaphylaxis [192]. The allergenicity of parvalbumin in bony fish is significantly higher in white muscle than in dark muscle. Its content also varies significantly between fish species. Parvalbumin levels are relatively low in tuna, ˂0.05 mg/g. However, in other common fish, its amount can be much higher: in mackerel meat, 30.7 mg/g; in salmon, trout, and cod, 12.5 mg/g; and in carp, herring, and redfish, >2.5 mg/g [314]. Physical and chemical food processing can change the allergenicity of fish through degradation or oligomerization of parvalbumins [315]. According to European Union (EU) regulations, food producers are required to declare the presence of 14 food groups classified as potentially allergenic, including fish, crustaceans, and mollusks, and to distinguish them from the list of other ingredients [225].

Other fish allergens considered to be of minor importance include β-enolase, aldolase, collagen, tropomyosin and vitellogenin [314,316]. Enolases (50 kDa) and aldolases (40 kDa) have been purified and identified as new allergens in freshwater fish species (bream, tilapia) [317] and marine fish species (*Atlantic cod*, *Atlantic salmon*, *Yellowfin tuna*) [314]. Enolases and aldolases are found in fish muscles and participate in metabolic glycolysis (sugar degradation) during physiological energy production. They are thermally unstable. Currently, there are several β-enolases and aldolases officially recognized as allergens by the World Health Organization/International Union of Immunological Societies (WHO/IUIS) Allergen Nomenclature Database (http://allergen.org): β-enolases isolated from *Atlantic cod* (Gad m 2), striped catfish (Pan h 2), *Atlantic salmon* (Sal s 2), and yellowfin tuna (Thu a 2). Tropomyosin is a major allergen of crustaceans and mollusks, but its allergenicity was first confirmed in 2013 in a freshwater fish species, tilapia (*Oreochromis mossambicus*) [318], and subsequently in marine fish species such as albacore tuna, cod, and swordfish [319]. It exhibits high thermal stability during heat treatment (boiling, steaming, frying, baking) without losing its allergenicity, thus posing a serious health risk to consumers [320,321].

Lipid oxidation, protein degradation, enzymatic reactions, and microbial growth in fish easily contribute to the deterioration of their flavor, aroma, and textural characteristics [322,323,324]. The umami flavor is a key determinant of fish preference among consumers and producers [325]. Typically, undesirable flavors that impart an unpleasant odor to freshwater fish meat include muddy, fishy, grassy, and musty. Their development is thought to stem from several sources [323,324]. The muddy odor results from the presence of geosmin (GSM) and 2-methylisoborneol (MIB), which are produced by the algae *Aphanizomenon*, *Oscillatoria*, *Nostoc*, and *Anabaena*, as well as *Streptomyces bacteria*, including *S. griseus* and *S. coelicolor* [325,326,327]. The accumulation of these compounds in the tissues of freshwater fish is the main cause of reduced sensory quality, which consequently leads to consumer disapproval and economic losses in fish processing [324,326].

In comparison, marine fish emit less intense undesirable odors, reminiscent of a sea breeze [328]. The main volatile compounds that shape the smell of fish are alcohols and carbonyl compounds (aldehydes, ketones, carboxylic acids), which constitute over 90% [323]. Ultimately, the flavor and odor of fish and fish products are profiled by a mixture of several compounds (hexanal, heptanal, octanal, nonanal, 1-penten-3-ol, 1-octen-3-ol, acetic acid, butanoic acid, and others) [329]. Compared to freshwater fish, marine fish contain higher levels of unsaturated fatty acids (MUFA) and polyunsaturated fatty acids (PUFA), which give them a richer flavor [324]. However, lipid oxidation can produce many compounds that cause undesirable aromas. Lipid oxidation intensifies not only the fishy odor but also undesirable textural changes through the interaction of oxidation products with proteins [330]. Lipid oxidation in fish muscle is induced by several catalysts, including hemoglobin, iron, and lipoxygenases, which cause a strong fishy odor, while hemoglobin produces an intense oxidized oil odor [331].

High-temperature processing and treatment of fish can result in the formation of volatile acids, sulfur-containing compounds (including dimethyl sulfide), carbonyl compounds, and amines (methylamine, propylamine, and butylamine), which ultimately shape their flavor [332]. The main lipid oxidation products in fish are hydroperoxides, which are unstable and decompose into secondary oxidation products, low-molecular-weight compounds—aldehydes, ketones, and carboxylic acids. These are the carriers of unpleasant odors characteristic of oxidative degradation. Some aldehydes include hexanal and olefin aldehydes: (E,E)-2,4-heptadienal, 2,4-decadienal, 2-hexenal, and (E,Z)-2,6-nonadienal are formed by the oxidative degradation of unsaturated fatty acids, which are typically considered the main volatile compounds responsible for a strong fishy odor [316]. Improper processing and storage of fish can lead to the formation of compounds such as trimethylamine (TMA), dimethylamine (DMA), hexanal (Caproic aldehyde), (E,E)-2,4-decadienal, (E,E)-2,4-heptadienal, heptanal, acetic acid, and butyric acid, which are responsible for the very unpleasant, rotten odor of fish [323]. The formation of unpleasant odors, intensifying the undesirable taste of fish, contributes to their disqualification from circulation, processing, and culinary use. Therefore, from a technological and consumer protection point of view, it is important to protect the raw material from external factors (oxygen, light, high temperature) and to process it into semi-finished and finished products as quickly as possible.

Overall, the use of freshwater fish biomass in functional foods should be evaluated through a risk–benefit framework. The recovery of proteins, peptides, lipids, collagen, polysaccharides, vitamins, and minerals may improve resource efficiency and provide nutritionally valuable ingredients. However, these benefits are relevant only when the recovered fraction meets food-grade requirements for heavy metals, microbial quality, biogenic amines, allergenic proteins, oxidation products, and sensory acceptability. Safety evaluation should consequently follow the entire production chain, from the origin and anatomical selection of the raw material through extraction and purification to the final enriched food and its intended level of consumption.

## 6. Conclusions

In summary, freshwater fish represent an important, yet still underutilized, source of bioactive compounds for food enrichment. Carp is particularly important in this context. Due to its scale of production, regional availability, and economic importance in many European countries, it can serve as a model raw material for the development of fish-derived functional ingredients. Carp meat and its processing byproducts, such as skins, scales, bones, heads, and viscera, can be a potential source of proteins, bioactive peptides, collagen, gelatin, lipids, minerals, and other compounds with nutritional and technological value. Their use aligns with the development of functional foods and the principles of a circular economy, reducing raw material losses and increasing the added value of fish processing.

At the same time, the current state of knowledge indicates significant research gaps. There is still a lack of sufficient application studies on ingredients obtained directly from carp and their behavior in real food matrices. Stability during processing and storage, the impact on product sensory characteristics, bioavailability, and long-term safety remain particularly poorly understood. Further optimization of extraction, purification, and stabilization methods is also necessary to ensure that the obtained fractions meet quality and regulatory requirements for food ingredients. Therefore, future research should focus not only on identifying bioactive compounds of the raw material but primarily on their functionality, process scalability, and potential for practical application in food products. This approach could contribute to a more comprehensive use of carp as a strategic freshwater raw material for innovative, sustainable functional foods.

## Figures and Tables

**Table 2:** Fatty acid composition [%] and fat content [%] in selected species of freshwater fish [23,85,98,99,100,101,102,103,104,105,106,107,108].

Species	ΣSFA	ΣMUFA	ΣPUFA	Σn-3PUFA	Σn-6PUFA	Σn-3HUFA	EPA+DHA	Fat
Rainbow trout	21.01–26.27	29.37–43.17	30.59–44.36	10.55–29.33	15.03–27.06	9.19–20.26	5.01–17.97	2.06–11.41
Common carp	22.45–28.49	36.50–52.71	18.75–35.01	9.26–19.91	9.18–15.10	5.78–17.57	0.84–7.18	0.68–6.48
Wels catfish	22.25–24.23	37.36–41.61	28.86–34.61	24.48–20.10	11.13–8.77	15.75–22.39	13.12–13.26	0.84–3.31
Perch	24.61–37.40	13.68–41.21	34.18–51.96	20.58–39.94	10.50–15.90	15.62–25.75	14.65–37.70	0.35–0.89
Bream	29.90–35.00	14.13–39.70	27.90–52.93	16.10–37.46	15.48–11.3	10.97–35.00	10.00–30.14	0.12–1.03
Pikeperch	21.04–22.10	40.70–22.77	38.26–50.05	20.70–38.80	11.26–17.55	16.99–33.45	15.63–33.45	0.79–1.11
Northern pike	26.70–36.50	13.95–22.35	49.02–53.30	34.61–39.10	10.20–16.33	31.72–36.47	28.73–33.55	0.56–0.75
Roach	29.61–35.40	13.79–47.64	19.96–51.82	8.19–38.13	9.60–17.47	5.15–36.16	4.49–35.11	0.64–0.94
Northern whitefish	22.49–29.70	28.30–45.22	32.39–43.40	21.86–38.17	6.50–10.43	19.34–36.40	17.83–36.38	9.82–13.87
Silver carp	25.22–37.70	25.60–44.61	24.85–38.90	16.70–18.40	6.45–14.50	10.06–22.50	8.19–19.70	1.20–5.78
Burbot	32.30–45.90	32.00–32.60	21.50–32.36	12.70–30.19	2.17–8.60	10.16–24.47	10.05–23.60	0.80–1.61
Bighead carp	27.00–36.74	21.80–44.82	18.45–36.99	13.32–23.00	2.59–11.40	8.85–13.74	7.18–15.60	1.50–8.20

**Table 4:** Content of macroelements and microelements per 100 g of meat of fresh freshwater fish [213,223,224,225,226].

Ingredient	Carp (*Cyprinus carpio*)	Rainbow Trout (*Oncorhynchus mykiss*)	Pike (*Esox lucius*)
	mg/100 g		
K	333–387	370–420	259–320
P	215–415	220–245	210–230
Ca	10–41	12–18	20–57
Mg	12–29	20–25	18–27
Na	35–49	31–52	39–50
Fe	0.70–1.24	0.50–0.80	0.50–0.60
Zn	1.00–1.48	0.48–0.60	0.66–0.90
Sn	0.01–0.02	0.01–0.04	0.02–0.03
Cu	0.05–0.06	0.04–0.06	0.03–0.06
Mn	0.04–0.05	0.02–0.04	0.01–0.04

**Table 5:** Macronutrient and micronutrient content per 100 g of fresh marine fish [216,225,226].

Ingredient	Salmon (*Salmo salar*)	Cod (*Gadus morhua*)	Halibut (*Hippoglossus hippoglossus*)
	mg/100 g		
K	366–380	320–413	370–446
P	230–261	184–210	236–210
Ca	7–13	9–16	7–14
Mg	27–29	22–32	23–28
Na	51–75	54–72	60–68
Fe	0.38–1.00	0.38–0.50	0.20–0.60
Zn	0.39–0.90	0.45–0.70	0.36–0.40
Sn	0.03–0.05	0.03–0.04	0.04–0.05
Cu	0.04–0.06	0.03–0.05	0.02–0.04
Mn	0.01–0.02	0.01–0.02	0.01–0.02

## Data Availability

No new data were created or analyzed in this study. Data sharing is not applicable to this article.
